# Supplementary material for: Development and Validation of Nutrition and Food Safety Educational Material for Fish Processors in Nigeria
Source: Int J Environ Res Public Health. 2023 Mar 10;20(6):4891. doi: 10.3390/ijerph20064891 (PMC10049519; doi:10.3390/ijerph20064891)
Supplement: Supplementary file 1 [file ijerph-20-04891-s001.zip › Supplementary Material-Nutrition and Food Safety Edcational Material for Fish Processors.pdf]

FEED THE FUTURE
The U.S. Government's Global Hunger & Food Security Initiative

NUTRITION EDUCATION, FOOD SAFETY, AND SAFE FISH HANDLING PRACTICE GUIDE FOR FISH PROCESSORS IN NIGERIA

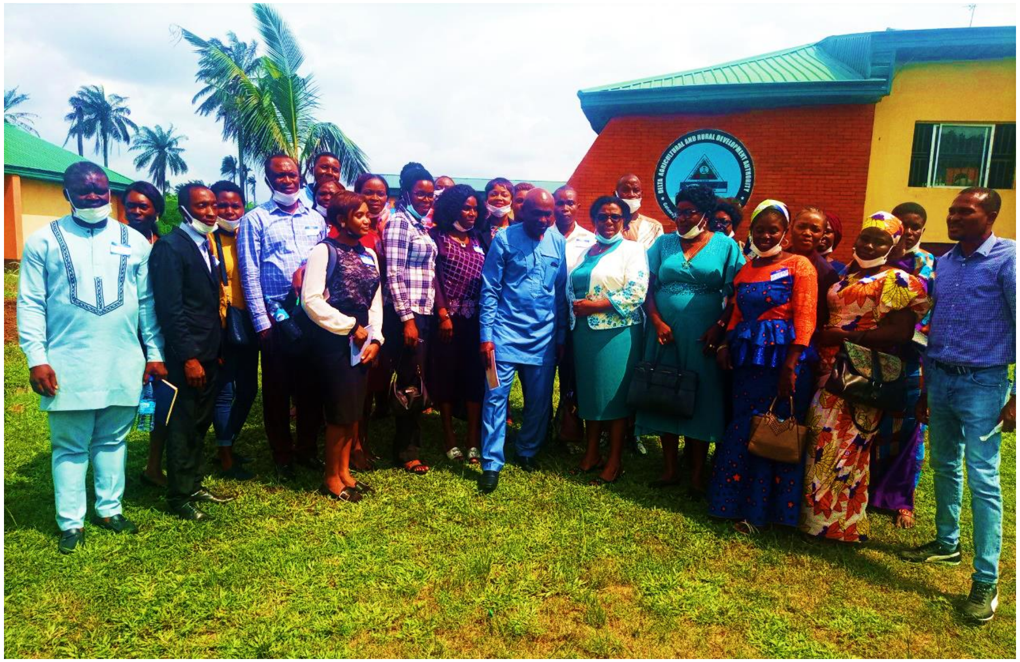

Feed the Future Innovation Lab for Fish Nourishing Nations Team

USAID FROM THE AMERICAN PEOPLE

WorldFish

MISSISSIPPI STATE UNIVERSITY™
GLOBAL CENTER FOR AQUATIC FOOD SECURITY

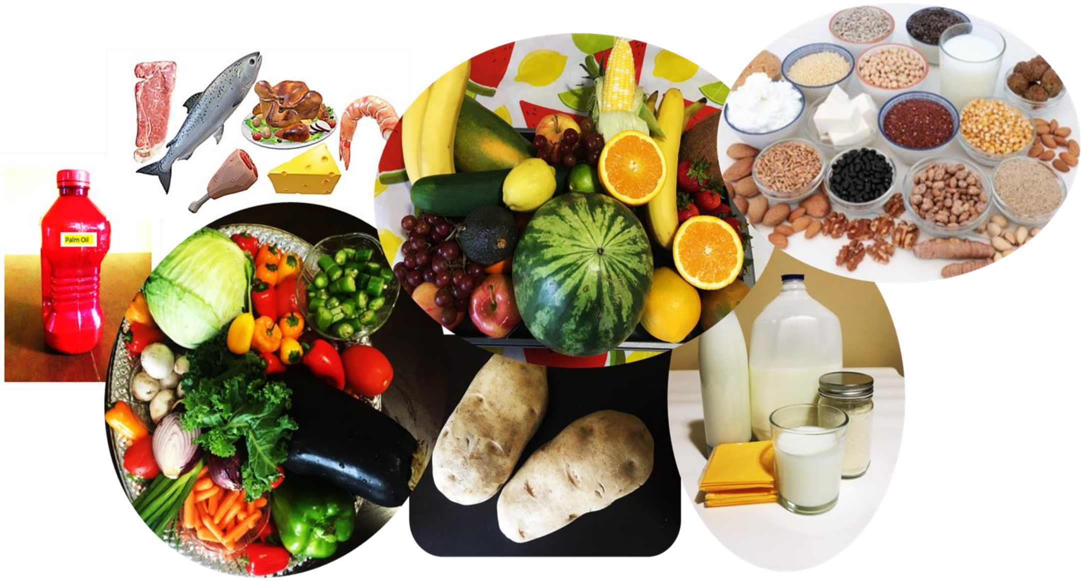

MODULE 1 NUTRITION EDUCATION

HEALTHY EATING

Nourishing Nations Project

August 2021/Delta State, Nigeria

HEALTHY DIET

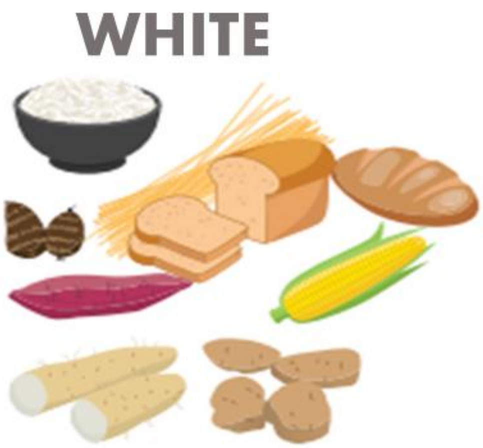

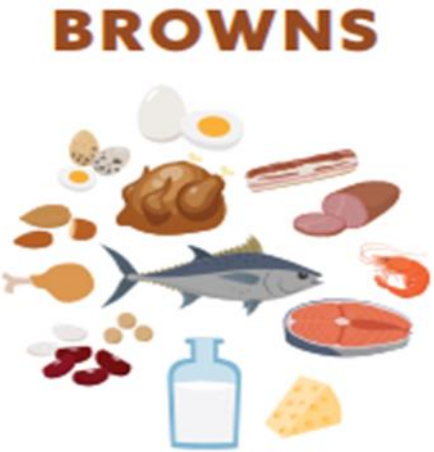

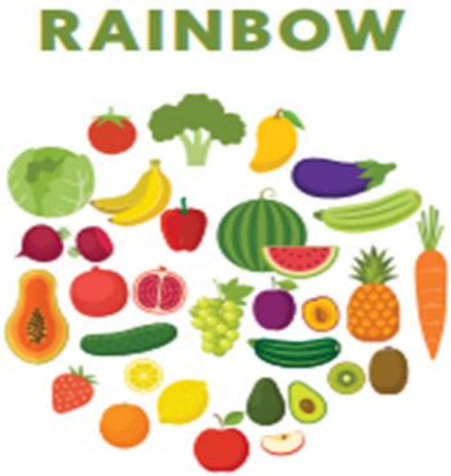

Home Garden Toolkit, World Vegetable Center.

EATING THE RIGHT PORTIONS

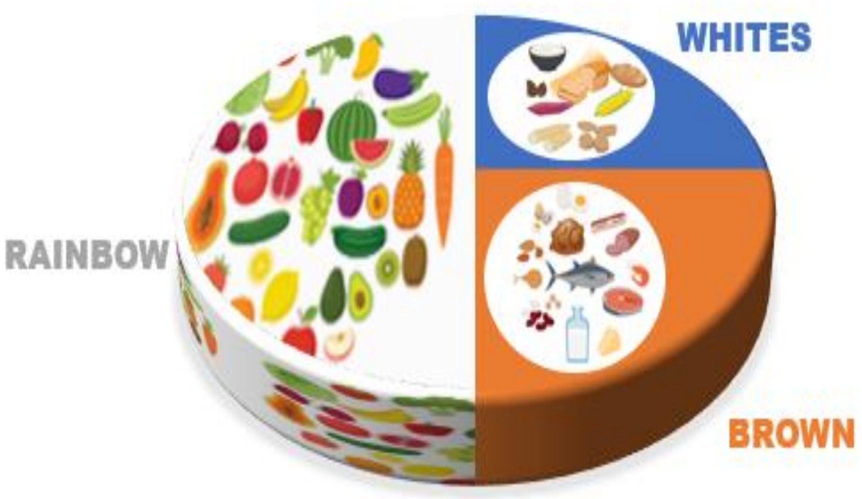

- **WHITE:** Grains, roots, and tubers (¼ of your meal)
- **BROWN:** Proteins, milk and milk product (¼ of your meal)
- **RAINBOW:** Fruits and Veggies (½ of your meal)
- **Diverse diet** are daily meals with a variety of foods from all the food colors.

Adegoye G.A 2022. Adapted from Home Garden Toolkit, World Vegetable Center.

WHITE, BROWN, AND RAINBOW FOODS

- **WHITE:** White foods are carbohydrates, and starch. They provide us with energy. Examples are rice, potatoes, cassava, cocoyam, yams, gari, maize, millet, and wheat.

- **BROWN:** Brown foods supply our bodies with proteins that build our muscles. Examples are fish, beef, pork, beans, eggs, chicken, seeds and nuts, tofu, and milk.

- **RAINBOW:** Rainbow foods (reds, oranges, yellows, greens, blues, purples) provide our bodies with the nutrients needed to fight disease and help our organs (eyes, heart, lungs, liver, and brain) function properly. Examples are mango, pawpaw, tomatoes, and spinach.

BENEFITS OF EATING RAINBOW FOODS:
FRUITS AND VEGETABLES

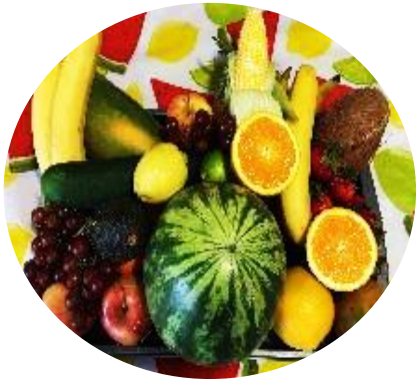

FRUITS
- Fruits are a good source of vitamin C, folic acid, and other nutrients. Fruit is good for pregnant women and children
- Eating fruit can help lower the risk of heart problems, high blood pressure and stroke
- Eating fruit may protect against certain types of cancers

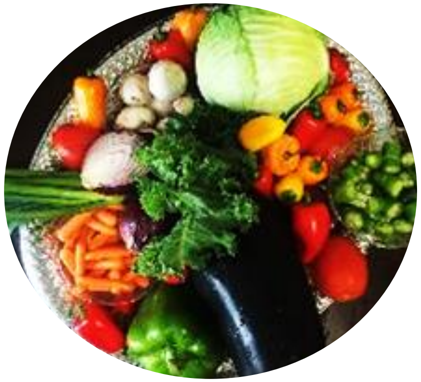

VEGETABLES
- Eating plenty of vegetables can help lower high blood sugar, high blood pressure, and risk of heart problems
- Vegetables are good for eyes, and skin, and keeps gums and teeth healthy
- Vegetables support proper digestion of food

BENEFITS OF EATING BROWN FOODS:

PROTEINS, MILK AND DAIRY

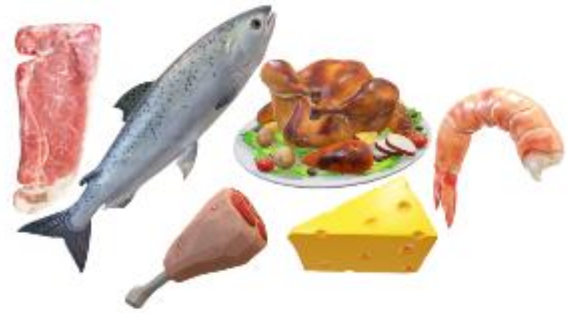

PROTEINS

- Proteins help in building body muscle and repairing worn out tissues.
- Omega 3 fatty acid in seafood can help to lower blood pressure

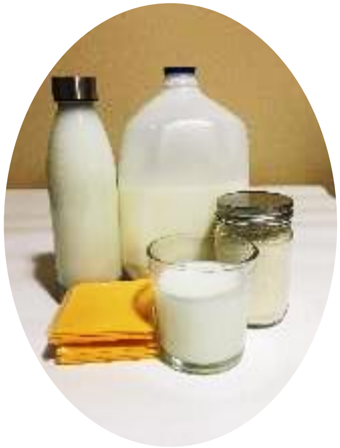

MILK AND MILK PRODUCTS (DAIRY)

- Good source of protein and it contains various vitamins and minerals that are important for healthy growth and development in children and pregnant women.

BENEFITS OF EATING WHITE FOODS:
GRAINS, ROOTS, AND TUBERS

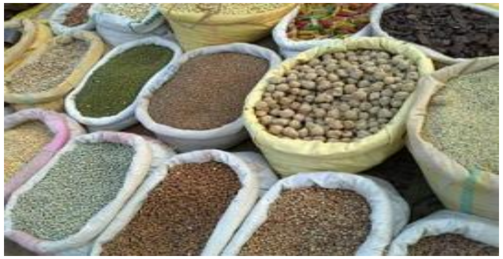

GRAINS
- Grains contain fiber, vitamins, and minerals
- Fiber in grains can lower the risk of colon cancer
- Fiber also helps in food digestion

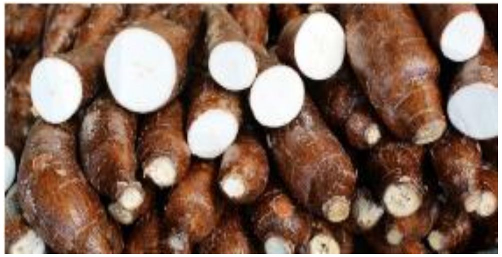

Source: modernfarmer.com

ROOTS AND TUBERS
- Root and tubers are rich in carbohydrates and starch. They provide energy
- They can help raise the sugar level in the blood when it is low. They also contain fiber.

BENEFITS OF BREASTFEEDING FOR INFANTS

HEALTHY EATING FOR INFANTS

1. Breastmilk contains all nutrients the baby needs for proper growth
2. Breastmilk provides natural protection to babies against diseases
3. Feeding only breastmilk for the first six months of life is healthy for both the baby and mother

CDC, Brown, J. E. 2016

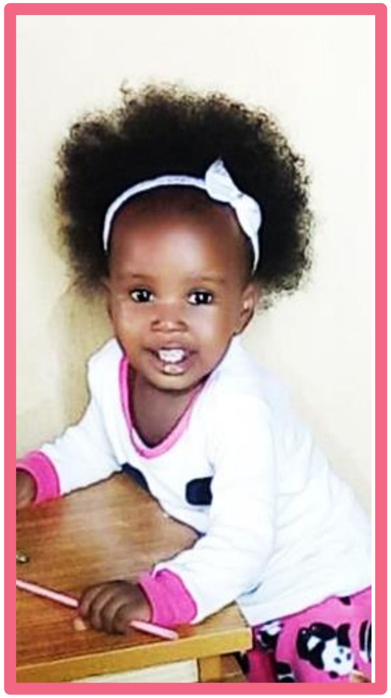

Photo by: Adegoye David. A

BENEFITS OF BREASTFEEDING TO MOTHERS

Breastfeeding can help to lower a mother's risk of:

1. high blood pressure
2. high blood sugar
3. breast cancer

CDC, Brown, J. E. 2016

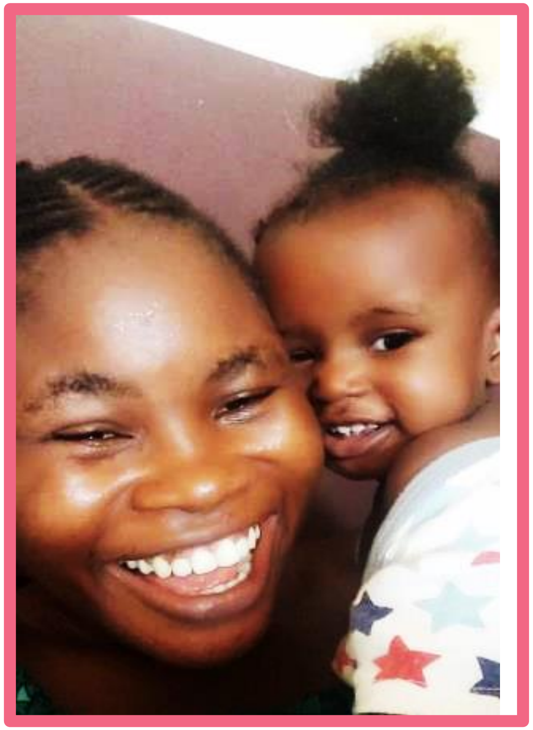

Photo by: Adegoye David. A

FEED THE FUTURE
The U.S. Government's Global Hunger & Food Security Initiative

HEALTHY EATING TIPS FOR WOMEN

- During pregnancy and breastfeeding, women need more energy
- Women's bodies change a lot during pregnancy and throughout breastfeeding to support the needs of the baby

1. Eat foods from healthy plate
2. Eat small extra meals
3. Eat when you feel hungry
4. Avoid processed and packaged foods
5. Also drink lots of water

USAID FROM THE AMERICAN PEOPLE
WorldFish
MISSISSIPPI STATE UNIVERSITY™ GLOBAL CENTER FOR AQUATIC FOOD SECURITY

EASY WAY TO MAKE HEALTHY MEAL

1. A healthy plate should contain one food from each color group every day.
2. A healthy plate has a lot of food variety to supply all the nutrients the body needs.
3. Change the foods you consume within each color group whenever possible.
4. A healthy plate is one-quarter white foods, one-quarter brown foods, and half rainbow foods.
5. Eat the right portion of each color at every meal. A colorful plate makes a healthy meal.
6. Eating the right mix of food can help you stay healthy.
7. Eat less processed, packaged, sugary, salty, and fried foods.

FEED THE FUTURE
The U.S. Government's Global Hunger & Food Security Initiative

MYPLATE FOR NIGERIA

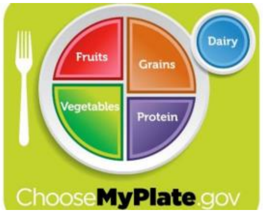

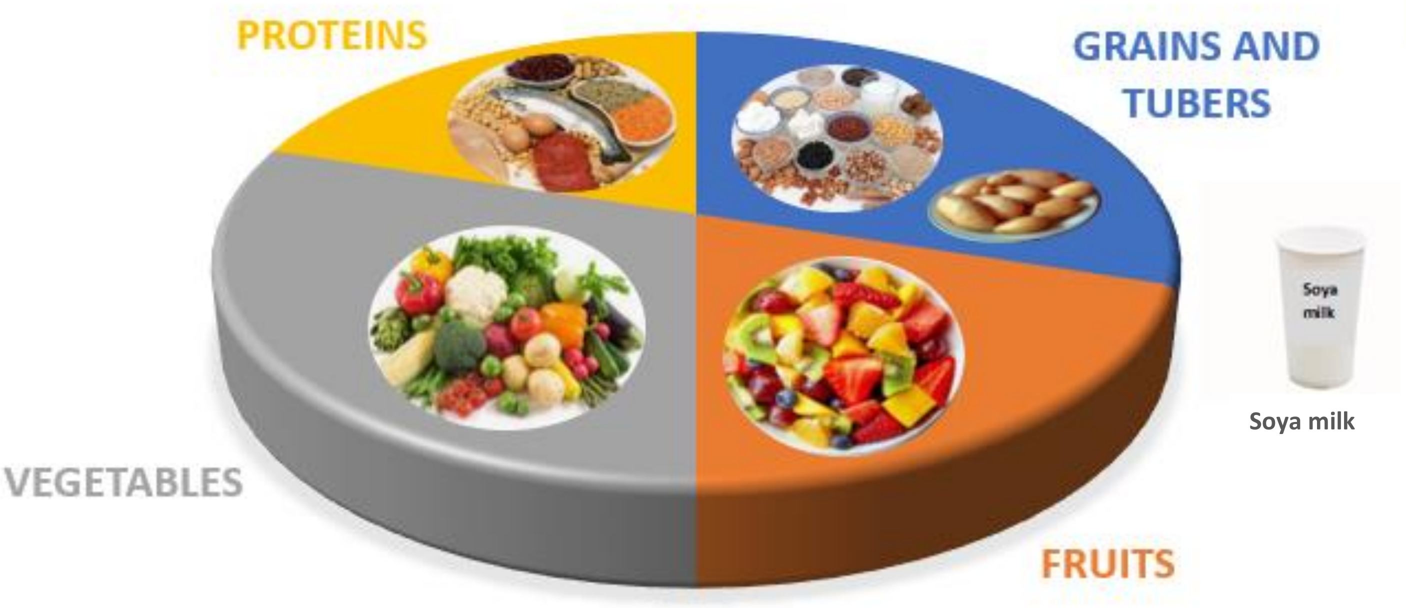

Adegoye G. A. 2022, Adapted from USDA MyPlate

USAID FROM THE AMERICAN PEOPLE

WorldFish

MISSISSIPPI STATE UNIVERSITY™ GLOBAL CENTER FOR AQUATIC FOOD SECURITY

FEED THE FUTURE
The U.S. Government's Global Hunger & Food Security Initiative

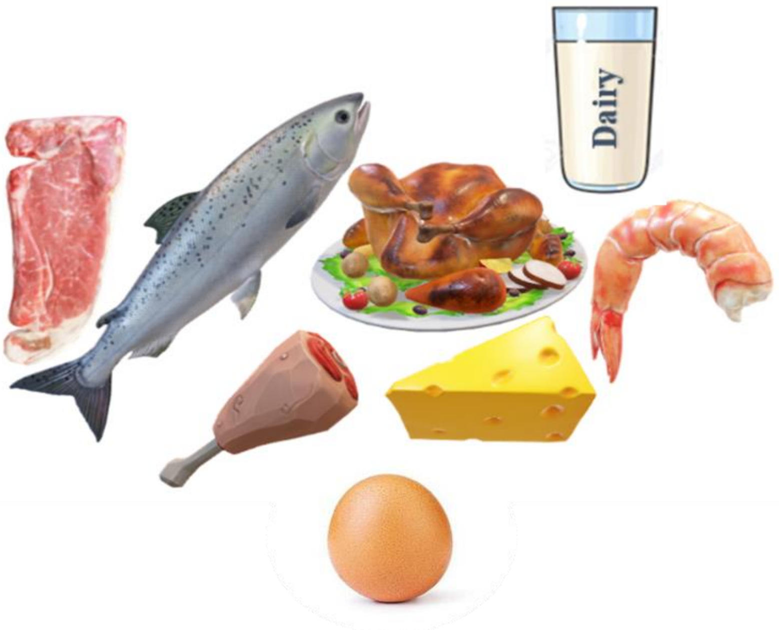

MODULE 2 ANIMAL SOURCE FOOD

ANIMAL SOURCE PROTEIN

Nourishing Nations Project

August 2021/Delta State, Nigeria

USAID FROM THE AMERICAN PEOPLE
WorldFish
MISSISSIPPI STATE UNIVERSITY™ GLOBAL CENTER FOR AQUATIC FOOD SECURITY

ANIMAL SOURCE PROTEIN

- Animal sources of protein come from animals and seafood.
- They are mostly in the brown food group.
- They supply nutrients that are important for children's growth.
- Examples are egg, fish, beef, chicken, milk, cheese, snail, crab, and other seafood.

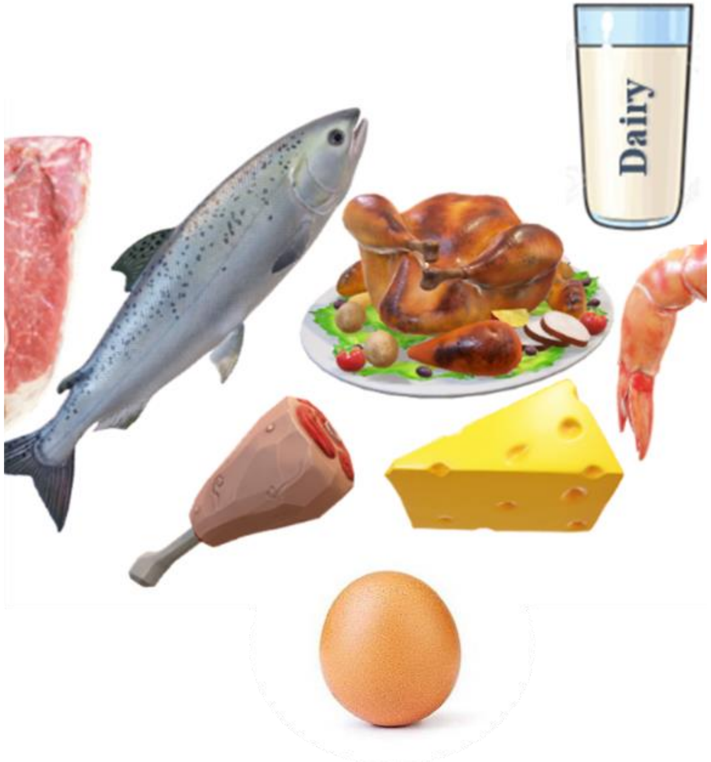

FISH

- Fish is one of the animal source foods
- It is rich in proteins
- It contains different kinds of vitamins and minerals
- Fish is cheaper than other animal source proteins
- It supplies nutrients important for normal growth to children and pregnant mothers

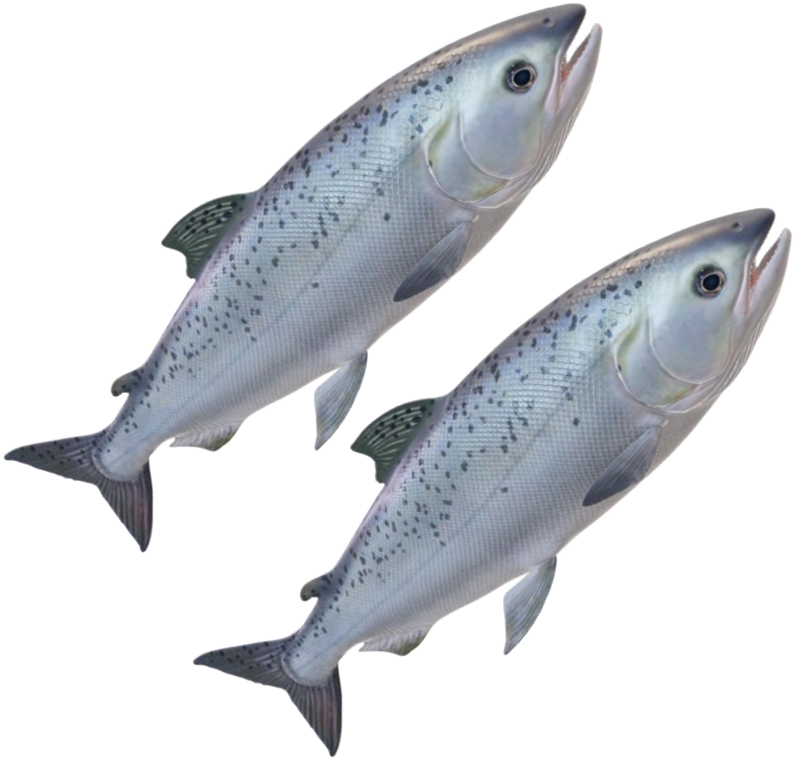

FEED THE FUTURE
The U.S. Government's Global Hunger & Food Security Initiative

MOTHER'S NUTRITION AND CHILD'S HEALTH

- Nutrition of the mother can affect her child's growth and development
- Nourished mother may deliver normal weight babies
- Unborn babies can be harmed by mother's poor nutrition
- The child may be sick even after birth
- Pregnant women, especially adolescent young girls, need more nutrients for normal growth and development of their child
- Eating different forms of animal source protein with other food groups will provide enough nutrient for the mother and the baby

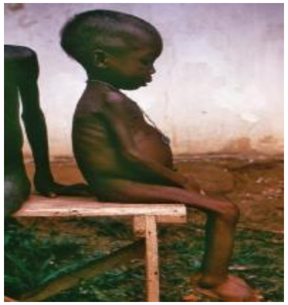

Photo by Dr. Lyle Conrad – Centers for Disease Control and Prevention, Atlanta, Georgia, USA Public Health Image Library (PHIL); ID: 6901.

USAID FROM THE AMERICAN PEOPLE
WorldFish
MISSISSIPPI STATE UNIVERSITY™ GLOBAL CENTER FOR AQUATIC FOOD SECURITY

FEED THE FUTURE

The U.S. Government's Global Hunger & Food Security Initiative

BENEFITS OF EATING FISH:

PREGNANT AND BREASTFEEDING MOTHERS

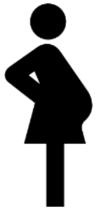

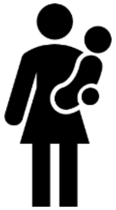

- Fish supplies nutrients (calcium, iron) for baby's growth and development during pregnancy and after birth
- Fish contains omega-3 fatty acids that are good for the mother's heart health
- Omega 3 fatty acid in fish can help with blood pressure during pregnancy

USAID FROM THE AMERICAN PEOPLE

WorldFish

MISSISSIPPI STATE UNIVERSITY™ GLOBAL CENTER FOR AQUATIC FOOD SECURITY

FEED THE FUTURE
The U.S. Government's Global Hunger & Food Security Initiative

BENEFITS OF EATING FISH:

INFANTS AND CHILDREN

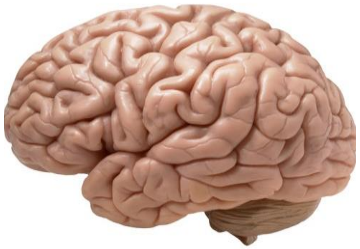

Brain development

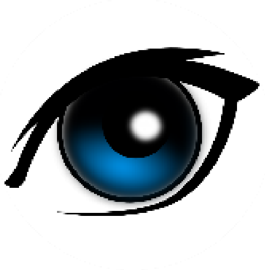

Good vision

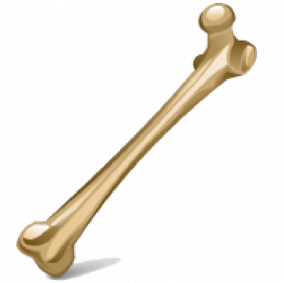

Strong bone

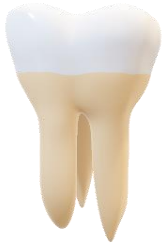

Strong teeth

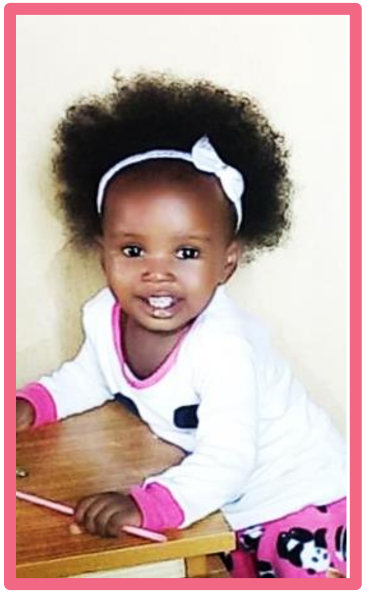

Normal growth

Photo: Adegoye David A.

USAID FROM THE AMERICAN PEOPLE

WorldFish

MISSISSIPPI STATE UNIVERSITY™ GLOBAL CENTER FOR AQUATIC FOOD SECURITY

GENERAL BENEFITS OF EATING FISH

Calcium supports bone and teeth health. It helps to prevent preterm delivery in pregnant women.

Vitamin D helps the body to get more calcium and other nutrients for normal growth.

Vitamin A protects the child from blindness due to lack of Vitamin A. It also helps to fight sickness and supports healthy growth.

Zinc supports normal growth (height) in children and prevents loss of weight from (diarrhea)

Iron is needed for brain development of an unborn child and supports safe delivery in pregnant women.

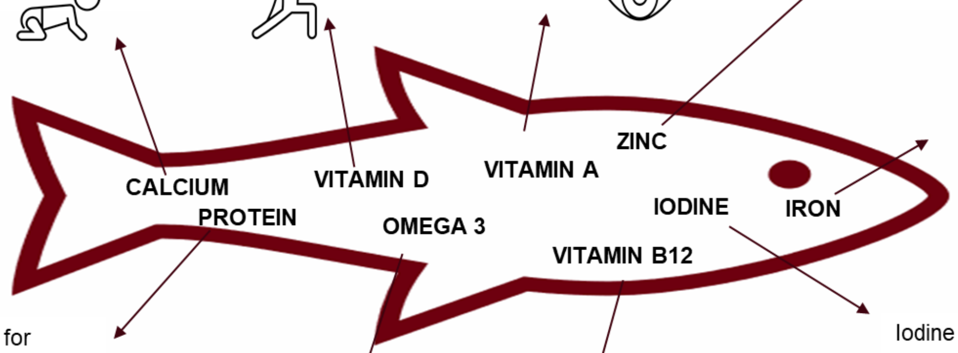

Protein is important for building muscles and helps in repairing worn out tissues. It also helps in the digestion of food.

Omega 3 fatty acids can support brain development in young children, and fight against sickness. It helps to protect the heart, reduce high blood pressure and the risk of stroke.

Vitamin B 12 helps to improve the red blood cells, and can help the body to fight against sickness

Iodine can help to prevent goiter in the neck region.

Adegoye G.A 2022

FEED THE FUTURE
The U.S. Government's Global Hunger & Food Security Initiative

MODIFIED FOOD FOR CHILDREN

OVER 6 MONTHS

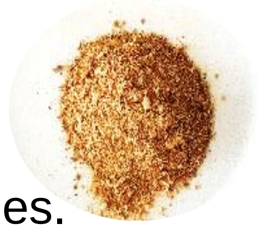

1. Introduce solid foods; rainbow, white and brown foods.
2. Small, dried fish can be pounded and mixed with stews or porridges.

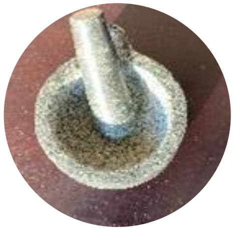

3. Mix paste fish with boiled vegetables, pulses, and stews.
4. Boil vegetables until they are soft and then mash.

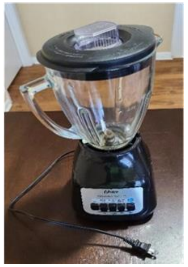

5. Boil beans until they are soft and then mash.
6. Fruits can be blended to make smoothies for young children.

USAID FROM THE AMERICAN PEOPLE

WorldFish

MISSISSIPPI STATE UNIVERSITY™
GLOBAL CENTER FOR AQUATIC FOOD SECURITY

FEED THE FUTURE
The U.S. Government's Global Hunger & Food Security Initiative

DIETARY RECOMMENDATION ON FISH EATING

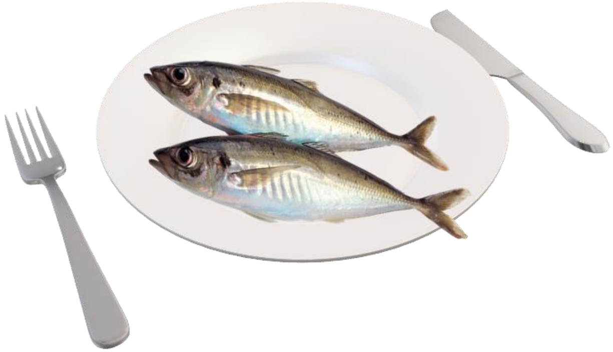

1. Eat fish at least 2-3 times a week
2. Serve 2-3 serving of fish a week to children
3. Eat a variety or different kinds of fish, if possible
4. Preferably serve small fish to children because they are more nutritious and safer.
5. Fish powder and paste can be mixed with other foods to provide extra nutrients
6. Serve fish with leafy green vegetables, and other rainbow foods.

USAID FROM THE AMERICAN PEOPLE

WorldFish

MISSISSIPPI STATE UNIVERSITY™ GLOBAL CENTER FOR AQUATIC FOOD SECURITY

FEED THE FUTURE
The U.S. Government's Global Hunger & Food Security Initiative

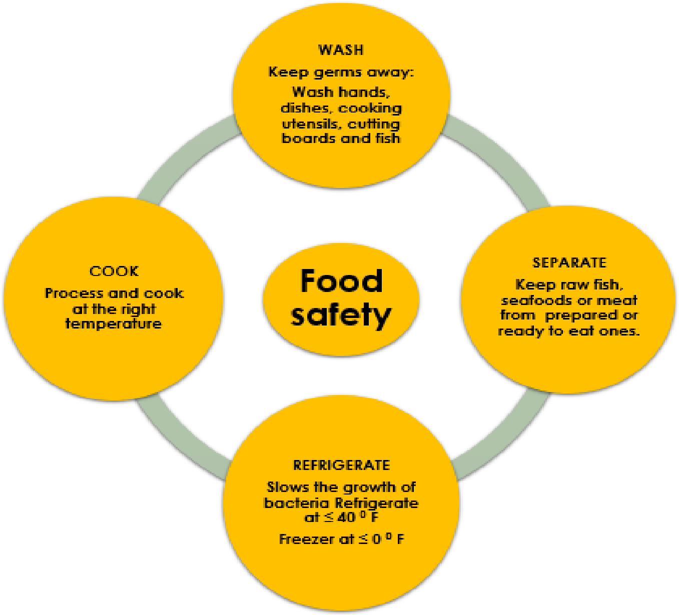

MODULE 3 FOOD SAFETY

FISH SAFETY AND HANDLING

Nourishing Nations Project

August 2021/Delta State, Nigeria

USAID FROM THE AMERICAN PEOPLE
WorldFish
MISSISSIPPI STATE UNIVERSITY™ GLOBAL CENTER FOR AQUATIC FOOD SECURITY

FEED THE FUTURE
The U.S. Government's Global Hunger & Food Security Initiative

SAFE FISH HANDLING AND PRACTICES

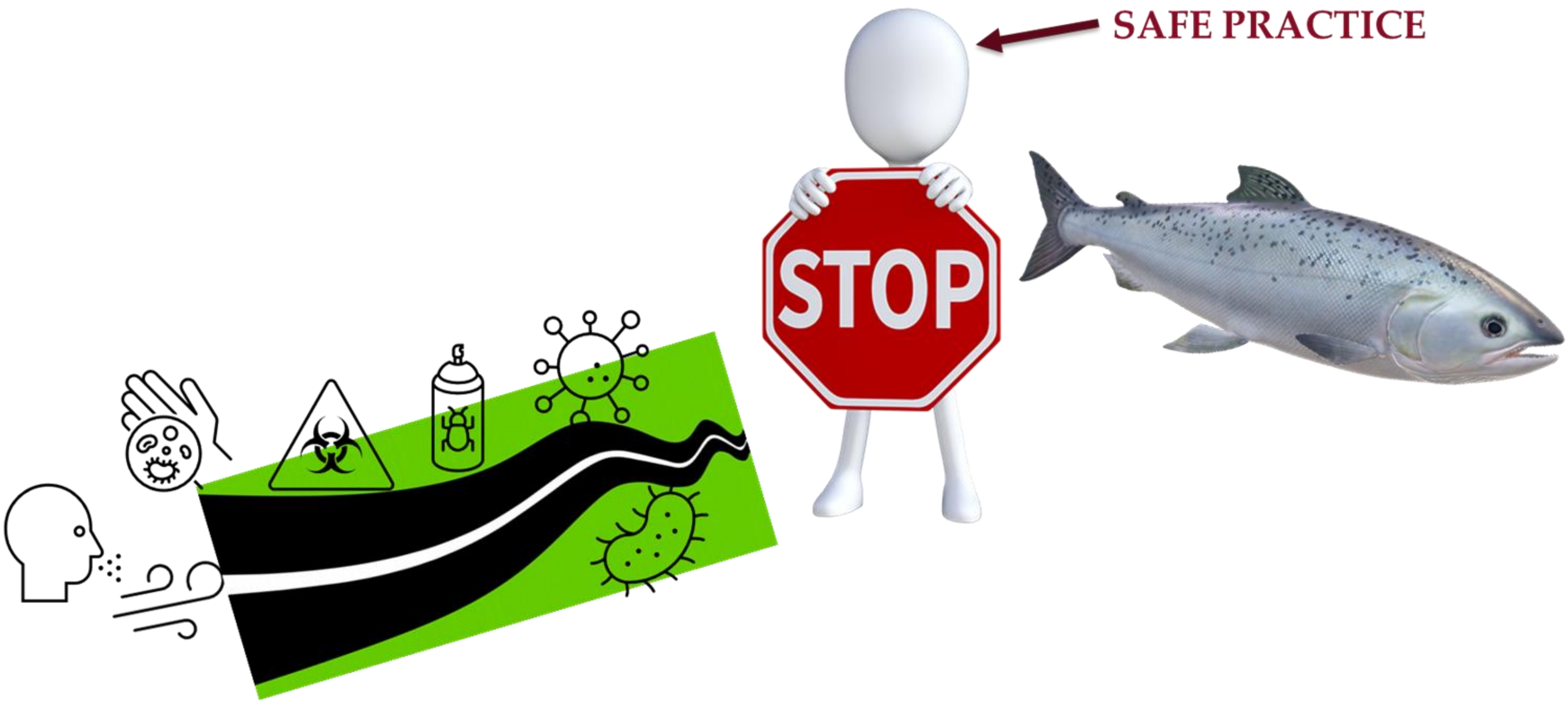

USAID
FROM THE AMERICAN PEOPLE

WorldFish

MISSISSIPPI STATE UNIVERSITY™
GLOBAL CENTER FOR
AQUATIC FOOD SECURITY

FEED THE FUTURE
The U.S. Government's Global Hunger & Food Security Initiative

KEYS TO FOOD SAFETY

WASH- wash your hands, utensils, cutting boards.

COOK- cook fish thoroughly to kill germs or bacteria.

SEPARATE- separate raw fish from processed or ready to eat food.

REFRIGERATE (Keep cool)- keep fish products at a low temperature. In the absence of a refrigerator use ice cubes or cold water.

USAID FROM THE AMERICAN PEOPLE

WorldFish

MISSISSIPPI STATE UNIVERSITY™ GLOBAL CENTER FOR AQUATIC FOOD SECURITY

KEEP RAW FISH UNDER A COLD TEMPERATURE

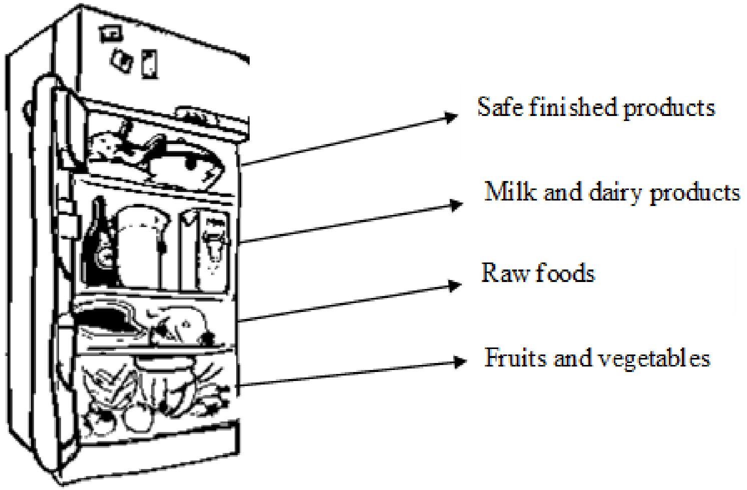

Keep raw fish and processed fish separately in a refrigerator

Adapted from FAO/PAHO-WHO 2014, *Food Handlers Manual*.

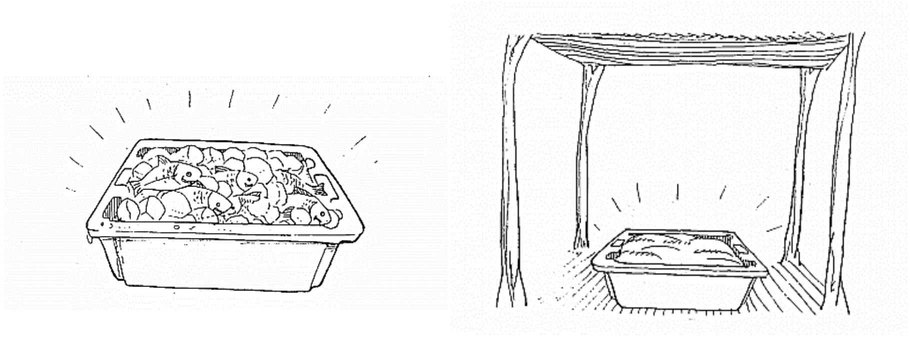

Keep raw fish in a container with ice cubes and enough clean seawater and/ or cover under shelter with a wet clean cloth if there is no ice.

Diei Y and Ndiaye O,1998.

FEED THE FUTURE

The U.S. Government's Global Hunger & Food Security Initiative

UNSAFE CONDITIONS THAT CAN SPOIL FISH

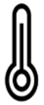

Temperature: raw fish spoils quickly when it is not under a cold or chilled condition.

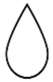

Moisture: Dried fish spoils when they stay too long under wet conditions.

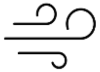

Air: human needs air (oxygen) to survive, so do food spoilage organisms.

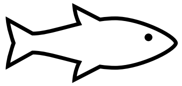

Food: food spoilage germs and other insect or animals feed on fish as food and cause spoilage

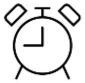

Time: fish spoils when it stays for long periods of time under unsafe conditions.

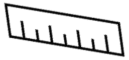

Acidity: neutral condition like water favors some food spoilage germs

Servsafe Manager, 2017

USAID FROM THE AMERICAN PEOPLE

WorldFish

MISSISSIPPI STATE UNIVERSITY™ GLOBAL CENTER FOR AQUATIC FOOD SECURITY

FEED THE FUTURE

The U.S. Government's Global Hunger & Food Security Initiative

WHY DO WE NEED TO PRACTICE FOOD SAFETY?

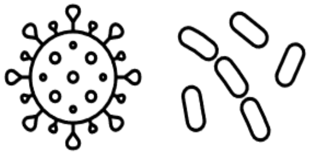

Keep germs or disease-causing agents out of food

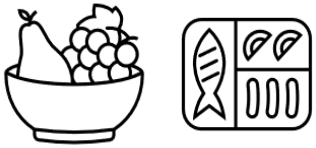

Enable safe and quality food free from germs

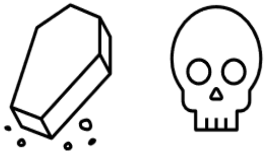

Prevent untimely death from food poisoning

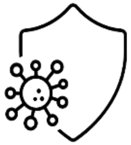

Protect us from getting sick (germs)

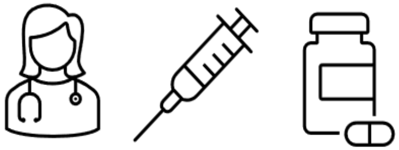

Reduce hospital bills for treatment

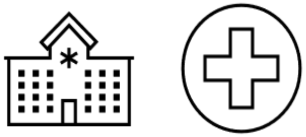

Reduce time spent sick in the hospital

USAID FROM THE AMERICAN PEOPLE

WorldFish

MISSISSIPPI STATE UNIVERSITY™ GLOBAL CENTER FOR AQUATIC FOOD SECURITY

FOOD-BORNE ILLNESSES

Food-borne illnesses or **illnesses** are caused by germs that can make us sick when they get into the food we eat or water that we drink.

How to know if you are suffering from food-borne sickness

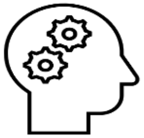

Headache

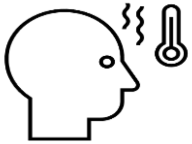

Fever

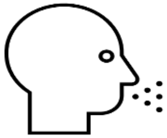

Vomiting

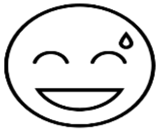

Sweating

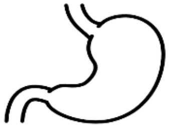

Stomach pain/discomfort

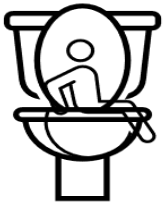

Stooling

SAFE PRACTICES

1. Do not handle fish if you suffer from these:

- Coughing
- Vomiting
- Stooling
- Fever
- Headache
- Stomach upset

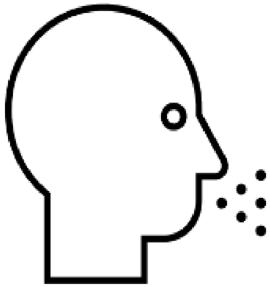

2. Visit hospital for treatment

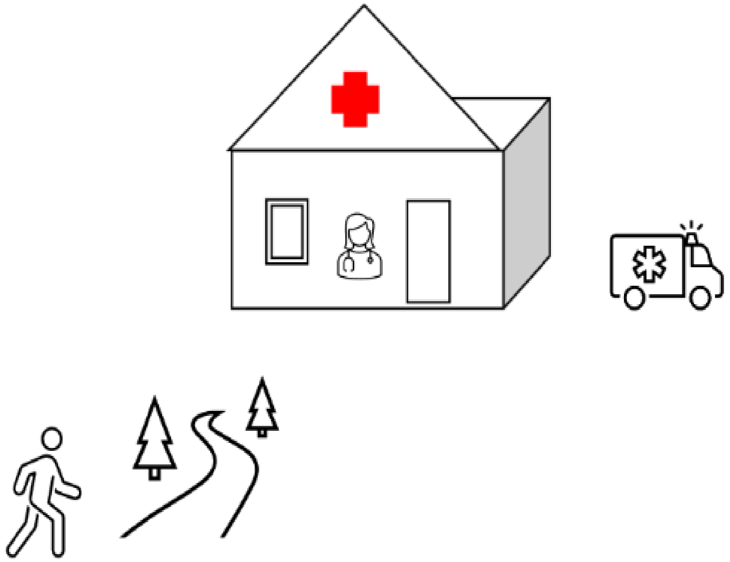

Adegoye et al, 2022

FEED THE FUTURE
The U.S. Government's Global Hunger & Food Security Initiative

HANDWASHING TIPS FOR FISH HANDLERS

1 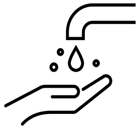 Wet your hands

2 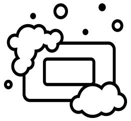 Apply soap & scrub for 20 seconds.

3 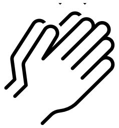 Rub palm to palm

4 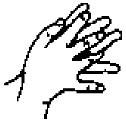 Rub back of your hands with fingers open

5 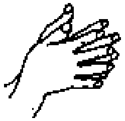 Rub palm to palm with fingers open

6 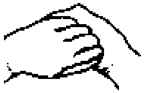 Rub back of fingers to palms with hands interlocked

7 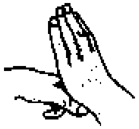 Rub the thumb

8 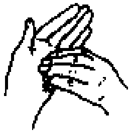 Wash the fingertips

9 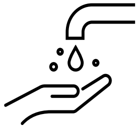 Rinse your hands with clean water

10 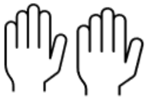 Air dry your hands. Now your hands are safe ☺

FAO/PAHO-WHO 2014

USAID FROM THE AMERICAN PEOPLE
WorldFish
MISSISSIPPI STATE UNIVERSITY™ GLOBAL CENTER FOR AQUATIC FOOD SECURITY

FEED THE FUTURE

The U.S. Government's Global Hunger & Food Security Initiative

WHEN TO WASH YOUR HANDS

Wash your hands

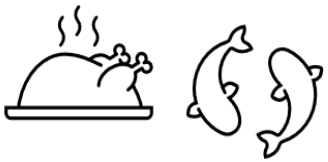

After handling raw fish or meat.

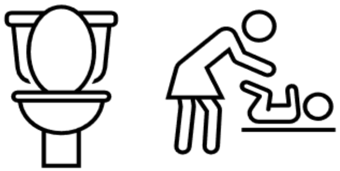

After using the toilet or changing baby's diaper

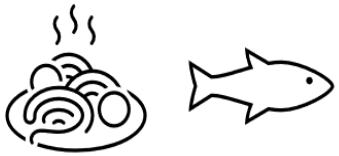

Before handling any food

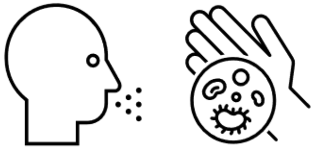

If you sneeze or cough into your hand

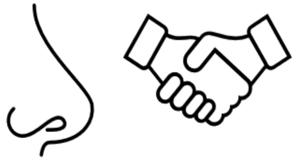

After picking your nose, scratching your body or shaking hands

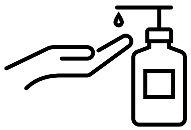

Hand sanitizer does not replace handwashing

Servsafe Manager, 2017

USAID FROM THE AMERICAN PEOPLE

WorldFish

MISSISSIPPI STATE UNIVERSITY™ GLOBAL CENTER FOR AQUATIC FOOD SECURITY

FEED THE FUTURE
The U.S. Government's Global Hunger & Food Security Initiative

PERSONAL HYGIENE OF FISH PROCESSORS

Wash your clothes, apron, and headwear regularly with soap and clean water.

Do not wear jewelry, ring, nail polish while handling fish

Bath at least once a day, especially after the day's job

Cut your fingernails to prevent germs from hiding under them

Brush your teeth at least once daily

USAID FROM THE AMERICAN PEOPLE

WorldFish

MISSISSIPPI STATE UNIVERSITY™ GLOBAL CENTER FOR AQUATIC FOOD SECURITY

FEED THE FUTURE
The U.S. Government's Global Hunger & Food Security Initiative

PERSONAL PROTECTIVE WEARS

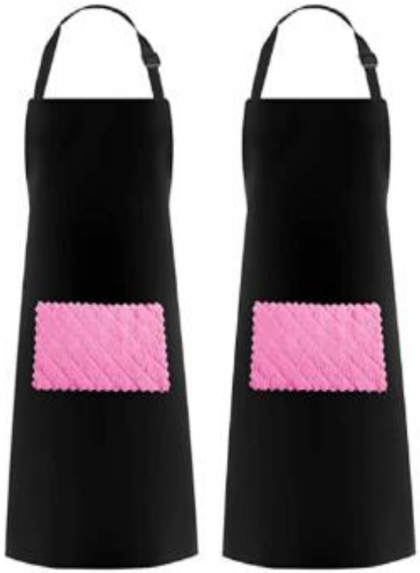
Picture from Amazon.com

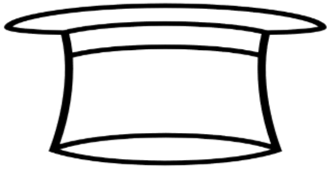

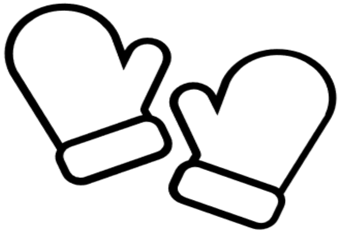

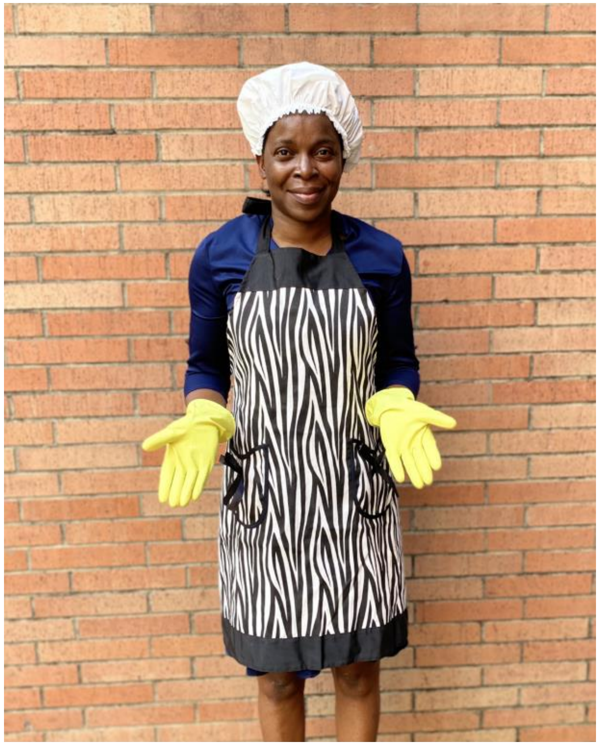
Photo by Alaina Dismuke

USAID FROM THE AMERICAN PEOPLE

WorldFish

MISSISSIPPI STATE UNIVERSITY™ GLOBAL CENTER FOR AQUATIC FOOD SECURITY

FEED THE FUTURE
The U.S. Government's Global Hunger & Food Security Initiative

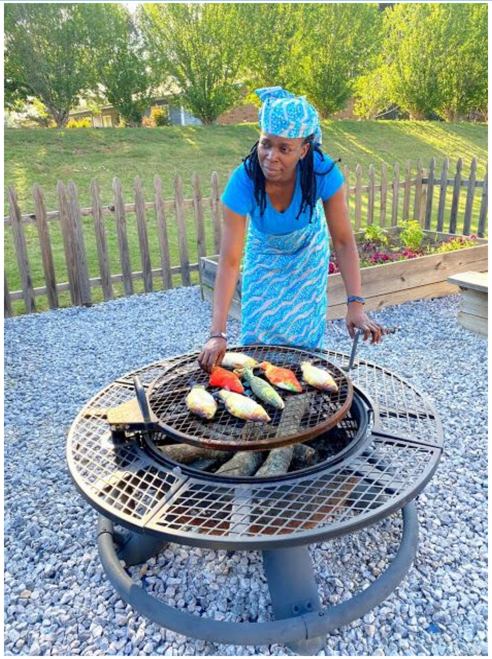

MODULE 4 FISH PROCESSING TECHNIQUES

FISH PROCESSING

Nourishing Nations Project

August 2021/Delta State, Nigeria

USAID FROM THE AMERICAN PEOPLE

WorldFish

MISSISSIPPI STATE UNIVERSITY GLOBAL CENTER FOR AQUATIC FOOD SECURITY

FISH PROCESSING METHODS

Sun-drying

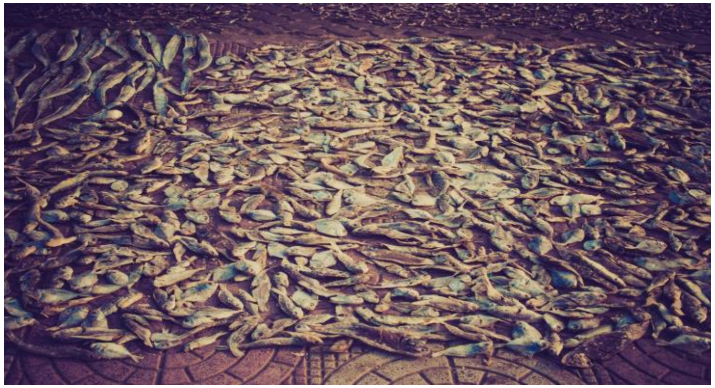

Source: Internet

Do not dry fish on the ground. It increases the risk of contact with dirt, germs, insects, and animals.

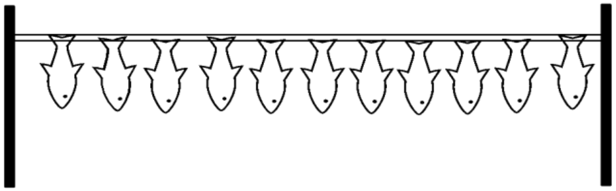

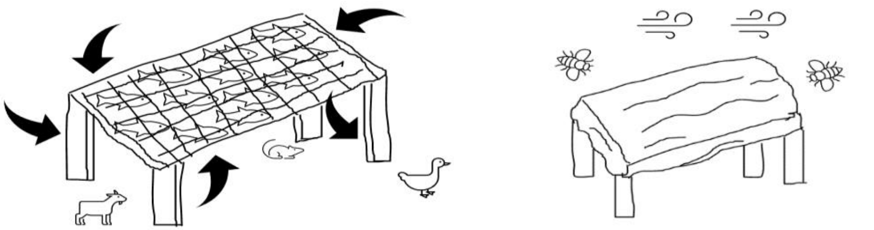

Dry fish by hanging or on a raised net tray to reduce exposure to germs, dirt, and stray animals. Cover with plastic or polythene nylon when necessary.

FISH PROCESSING METHODS

Salting

- Salting is a very good method to keep food spoiling germs away from food.
- It can be combined with other methods like smoking and drying.
- It slows down the growth of food spoiling germs
- Drying and salting is a good way to keep fish or animal protein for a longer time where there is no refrigeration or electricity.
- Two types of salting are: **Dry salting** (rub or sprinkle salt directly on the fish) and **wet salting** (soak fish in salty water).

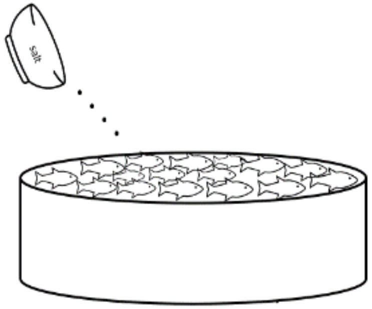

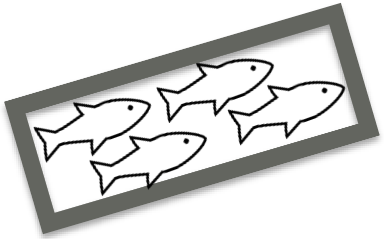

FISH PROCESSING METHODS

Smoking

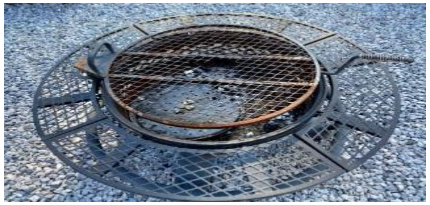

Metallic smoking kiln

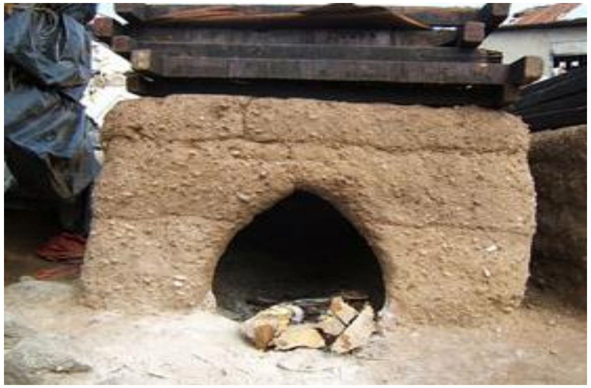

Traditional smoking mud kiln

Three processes or stages of fish smoking

- Cooking at a very high heat for 1-2 hours
- Drying at a low heat for 2 hours
- Smoking at a moderate heat for about 2 days

FEED THE FUTURE
The U.S. Government's Global Hunger & Food Security Initiative

MODERN METHODS OF FISH PROCESSING

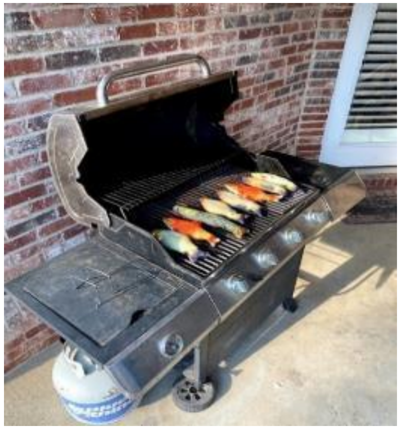
Gas Griller

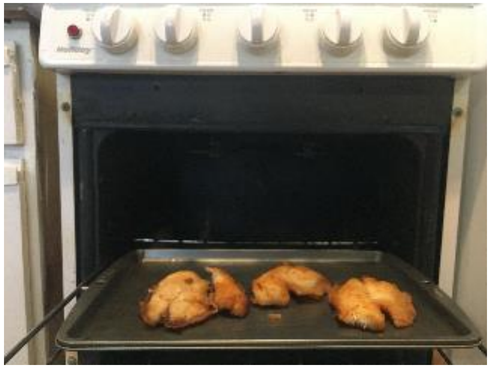
Modern or Electric Oven

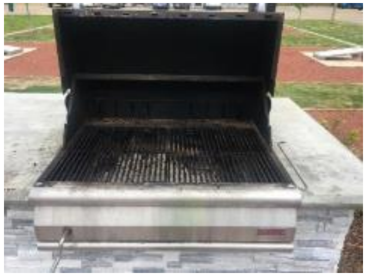
Modernized Charcoal Griller

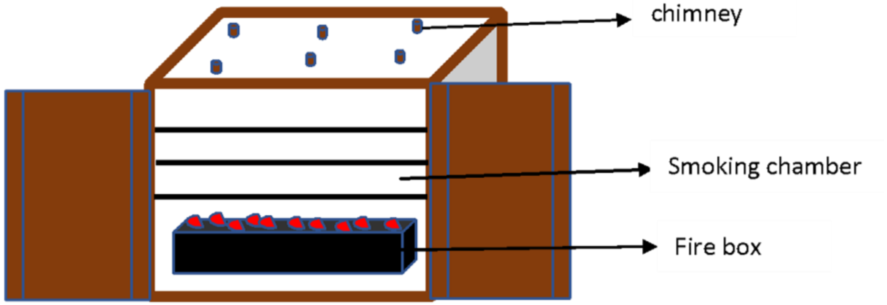

Modern Fish-smoking Kiln

USAID FROM THE AMERICAN PEOPLE

WorldFish

MISSISSIPPI STATE UNIVERSITY™ GLOBAL CENTER FOR AQUATIC FOOD SECURITY

MODERN METHODS OF FISH PROCESSING

Solar-drying

- Solar drying is a modern method of drying fish.
- Fish is placed and dried in a solar dryer and placed outside in the sun.
- It can be locally made and its easy to operate.
- It produces better quality dried fish product.

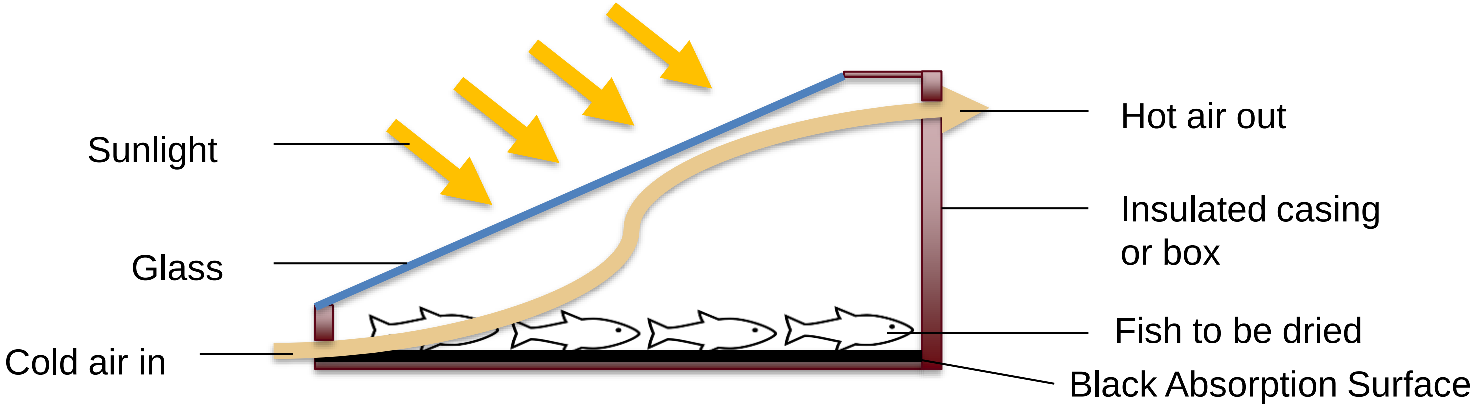

MODERN METHODS OF FISH PROCESSING

Freezing- chilling

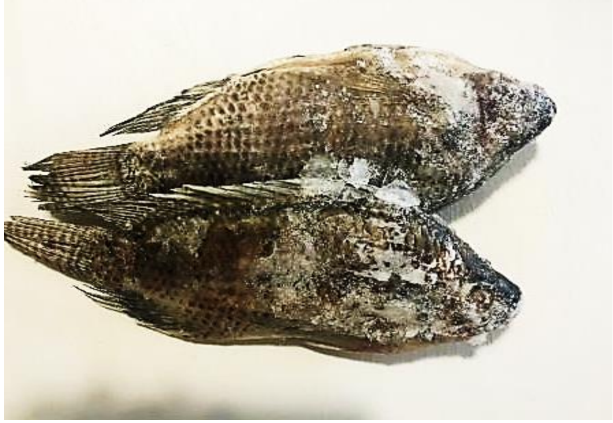

Limitations of freezing method

- Lack of electricity causes fish to spoil, smell bad, or loses nutrient as the icy fish melts. This may cause fish loss or waste and loss of profit.
- Freezers or refrigerators cost a lot of money
- Raw fish can be kept in a container with ice cubes or cooler, but electricity and freezer are needed to produce ice

LOCAL PROCESSED FISH PRODUCTS

Smoked fish: smoke can kill bacteria or germs and make fish stay in a safe condition longer.

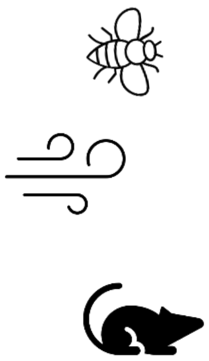

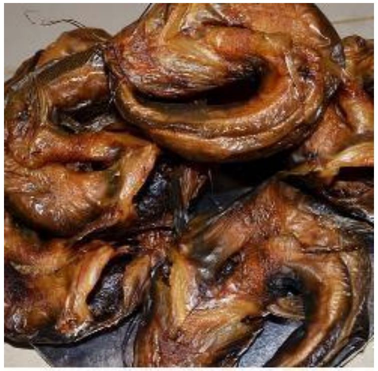

Photo by: Adegoye Grace A.

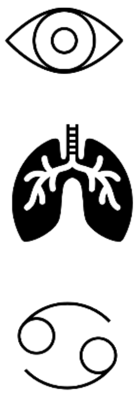

Attract flies and rodents.

Smoke can cause poor vision, poor breathing, and certain types of cancer.

Barbequed fish are grilled. They look nice and tastes good.

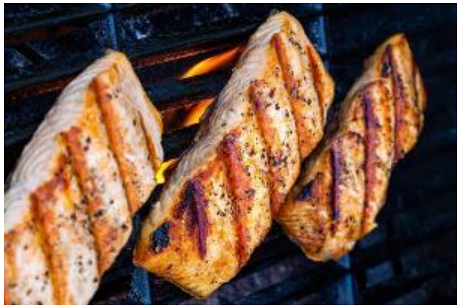

https://www.jessicagavin.com/grilled-salmon/

Barbequed fish may be exposed to dust and smoke

FEED THE FUTURE
The U.S. Government's Global Hunger & Food Security Initiative

LOCAL PROCESSED FISH PRODUCTS

Sundried fish

Removes water content from the fish to make it last longer without going bad.

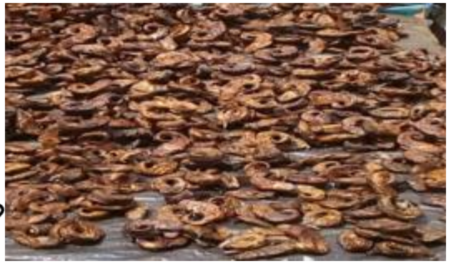

- Exposure to sand, dust, flies, pests, and rats
- Mold grows on dried fish when stored under wet (humid) conditions.

Fried fish has a nice taste, and smell. Some nutrients may be loss through frying.

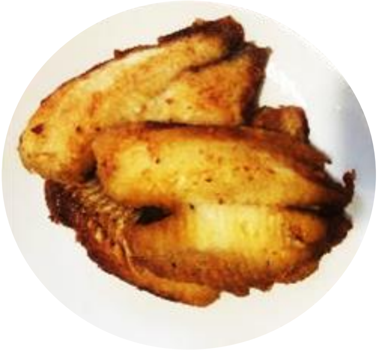

Fried fish attracts rats and cats.

Fried foods may increase the risk of heart disease and stroke. (American Heart Association)

Photos by Nuntah Joseph & Adegoye Grace A.

USAID FROM THE AMERICAN PEOPLE

WorldFish

MISSISSIPPI STATE UNIVERSITY™ GLOBAL CENTER FOR AQUATIC FOOD SECURITY

FEED THE FUTURE
The U.S. Government's Global Hunger & Food Security Initiative

MODERN PROCESSED FISH PRODUCTS

Powdered and Paste fish

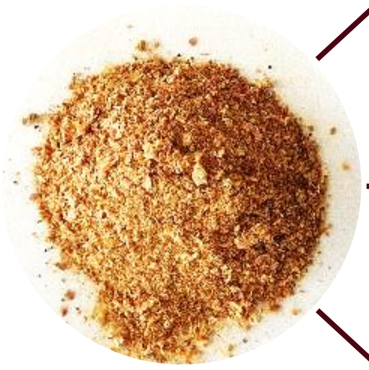

Nutrition

- Rich in calcium
- Rich in protein

Quality

- Can be measured
- Can be mixed safely in child's food

Business

- Can be nicely packaged
- Brings business growth and profit

USAID FROM THE AMERICAN PEOPLE

WorldFish

MISSISSIPPI STATE UNIVERSITY™ GLOBAL CENTER FOR AQUATIC FOOD SECURITY

FEED THE FUTURE
The U.S. Government's Global Hunger & Food Security Initiative

FISH PRODUCTS FROM MODERN METHODS

Canned fish

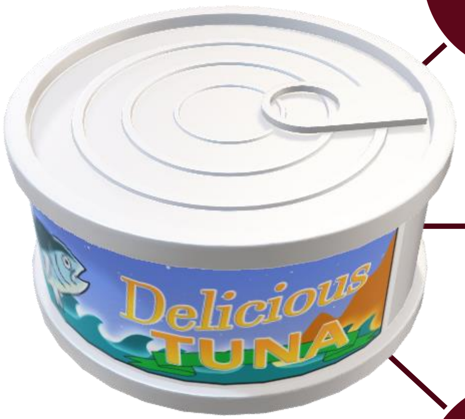

Nutrition

- Rich in calcium, iron, proteins.
- Supports child and pregnant women's nutrition

Quality and safety

- Free from dust, flies, and pests.
- Safe, nutritious and ready to eat

Business

- Safe fish for a longer time. Fish is available year round.
- Business growth and profit.

USAID FROM THE AMERICAN PEOPLE

WorldFish

MISSISSIPPI STATE UNIVERSITY™ GLOBAL CENTER FOR AQUATIC FOOD SECURITY

FEED THE FUTURE
The U.S. Government's Global Hunger & Food Security Initiative

FISH PROCESSING AND PROCEDURES

1

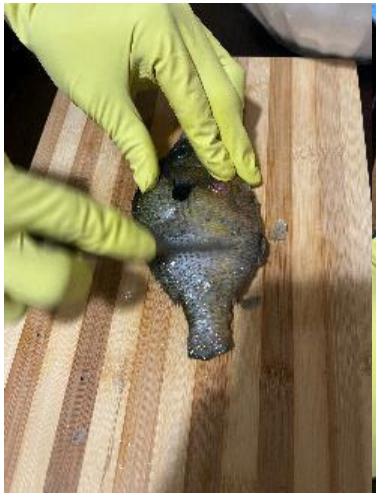

Remove scale

2

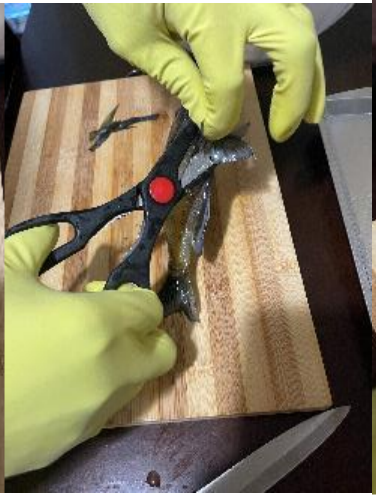

Remove fin

3

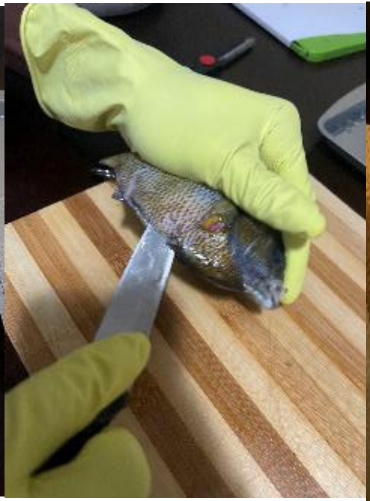

Remove intestine

4

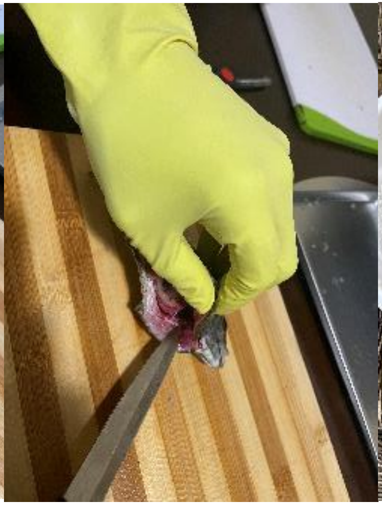

Remove gill

5

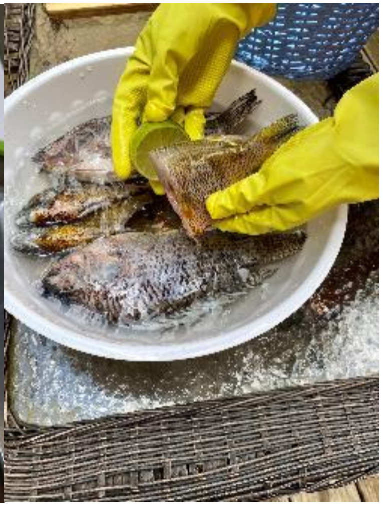

Cleaning-remove slime

USAID FROM THE AMERICAN PEOPLE

WorldFish

MISSISSIPPI STATE UNIVERSITY™ GLOBAL CENTER FOR AQUATIC FOOD SECURITY

FISH PROCESSING-CUTTING

Fish cutting: Cutting of the head, cutting into large or small pieces (chunks); splitting, filleting.

1. Fish splitting is cutting the fish open
2. Fish filleting: separating the flesh of the fish from the bone.

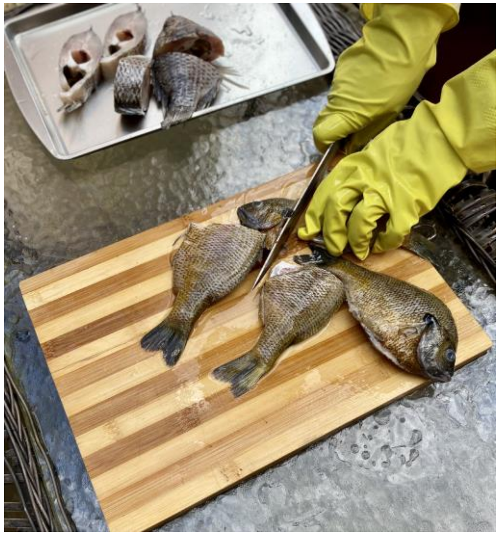

Cut the fish into sizes and shapes

Photo by Dismuke Alaina

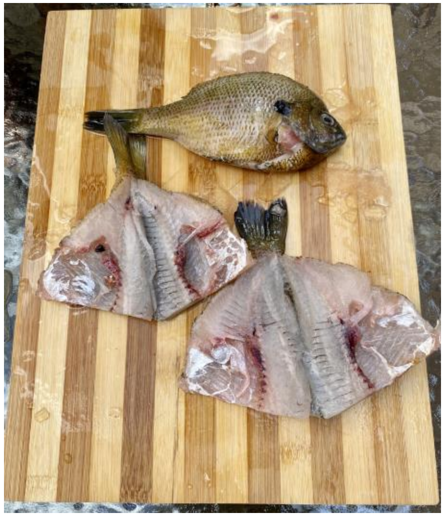

Fish Splitting

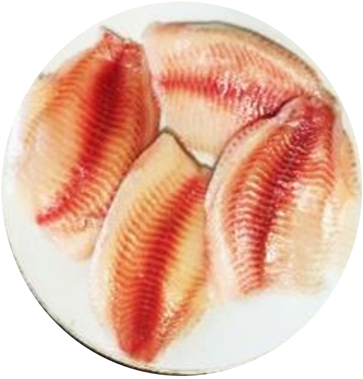

Fish fillet

FISH PROCESSING AND PREPARATION

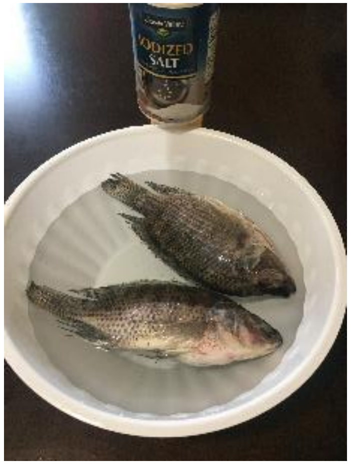

Soaking- wet salting

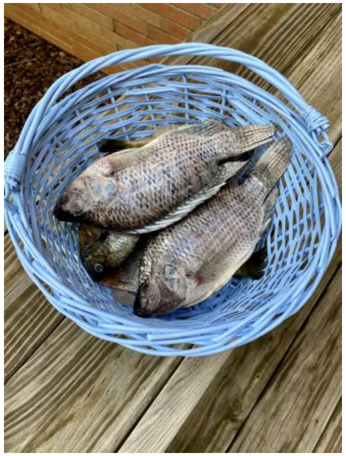

Sieving-Reduce water

Drying
- Solar drying
- Oven drying
- Hanging (Air drying)

Drying-remove water

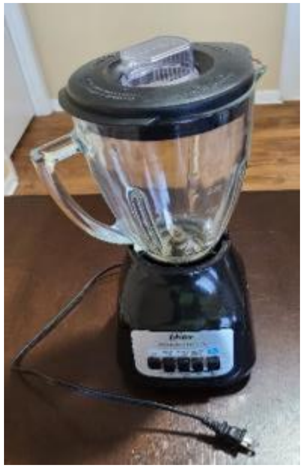

Grinding- blending

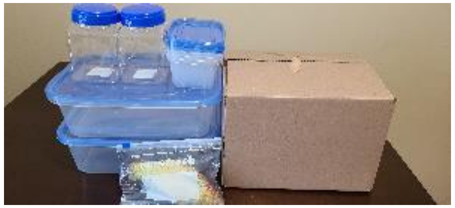

Packaging

FISH PROCESSING AND PROCEDURE

1. **Fermentation:** Raw fish is kept under a warm condition in the fermentation tank for a period ranging from a few hours to a week or weeks

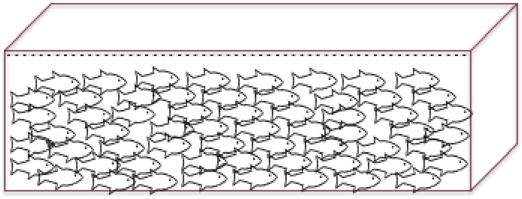

2. Pasting: Fish paste can be produced by fermentation and by grinding

i. After fermentation ->Rinse the fish with seawater or clean water- > Add salt as preservative-> Package

ii. Rinse raw fish in clean water

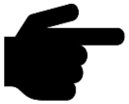

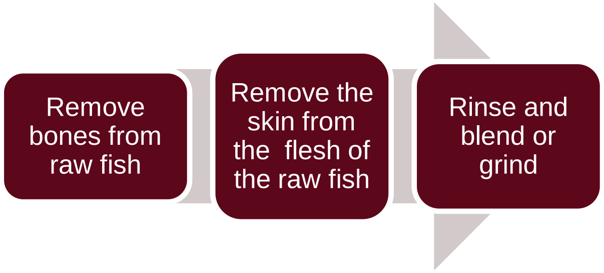

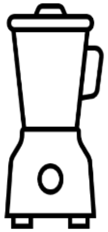

ADDITIONAL TIPS:

- Use quality wood for smoking to have a quality smoked fish product.
- Use hard and dry woods.
- Use quality charcoal for smoking
- Do not use rubber-like and painted woods for smoking.
- Do not use plastics, nylon, or rubbish for fish smoking

FEED THE FUTURE
The U.S. Government's Global Hunger & Food Security Initiative

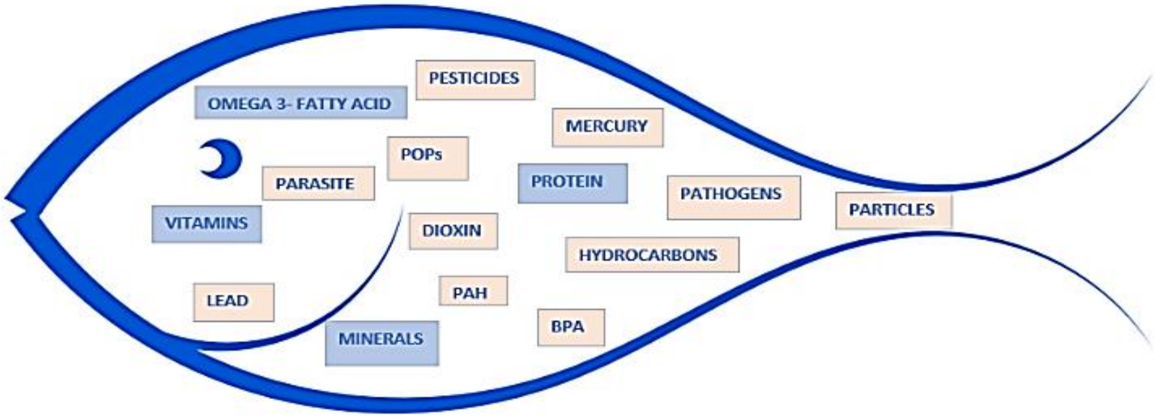

MODULE 5 FISH POISONING AND CONTAMINATION FISH CONTAMINATION

Nourishing Nations Project

August 2021/Delta State, Nigeria

USAID FROM THE AMERICAN PEOPLE

WorldFish

MISSISSIPPI STATE UNIVERSITY™ GLOBAL CENTER FOR AQUATIC FOOD SECURITY

FISH CONTAMINATION

Fingers (dirty hands containing germs)

Flies (insects) & foes (pests, rodents)

Fomites (dirty apron, handkerchief, napkins)

Fumes (gases or smoke, fog, etc.)

Forks (represents cutleries and cooking utensils)

Field (contaminated river or fish source)

Floor (dirty floor, dust, soil, sand, grit)

Fluids (dirty water, nose discharge, saliva, stool, urine, wound discharge, industrial waste)

Feces (human, animal, insect excreta)

Fahrenheit (Temperature at or above 40 °F. **"Danger Zone" (40-140 °F)**

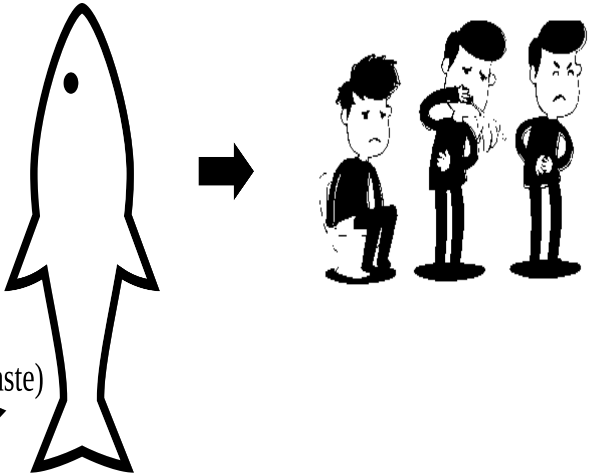

Adegoye G.A. 2022

WHAT ARE CONTAMINANTS

- **Contaminants** are any object, thing, or germs that can cause danger and harm us when present in our food or water.
- **Contamination** is when a harmful substance is present in food, drink, or water meant for eating or drinking.

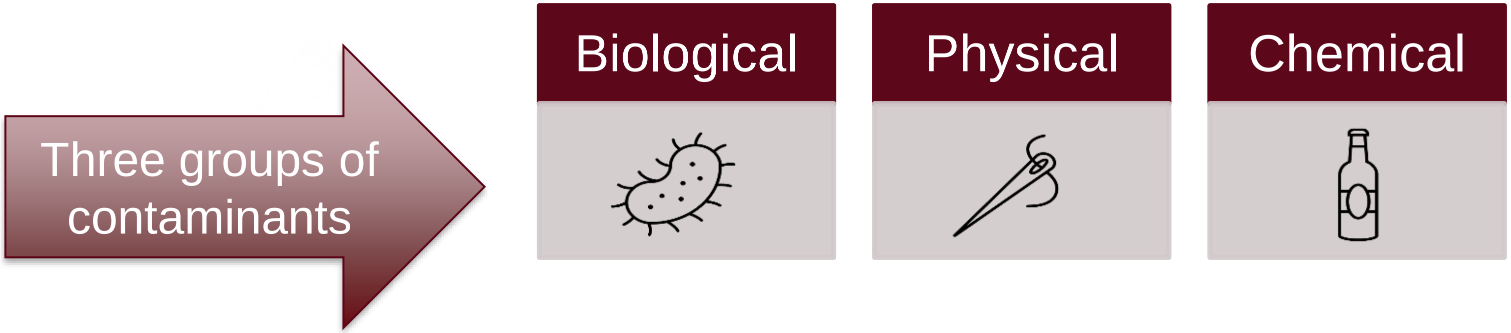

BIOLOGICAL CONTAMINANTS OR HAZARD

Biological contaminants or hazards in food are pathogenic organisms capable of causing foodborne illnesses or diseases and food spoilage. Pathogens or germs include bacteria, fungi, viruses, protozoa, prions. Some plants and seafood also carry harmful toxins. Mold on dried fish containing aflatoxin is also included in this group.

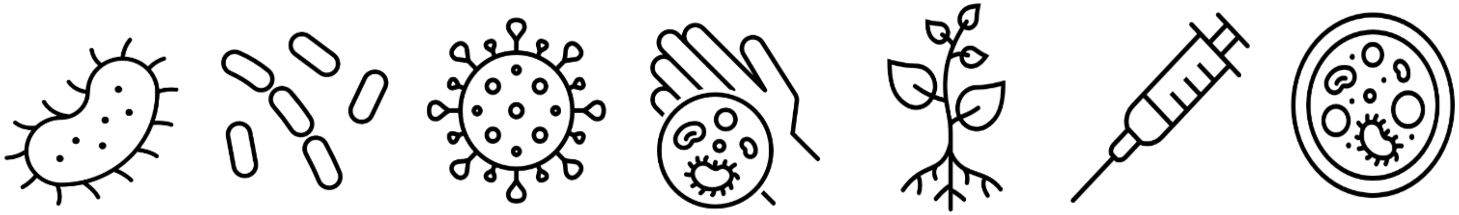

Adegoye, G.A, 2022

PHYSICAL CONTAMINANTS OR HAZARD

Physical contaminants or hazards are harmful objects or materials that can be seen, touched or felt. Examples are a piece of glass, wood, dust, dirt, sand, metal shavings from knives, a piece of bone, or sharp part of fish like the fin, pieces of plastic, stones, bandages, bag ties, hair strand, and other items used by the fish processor.

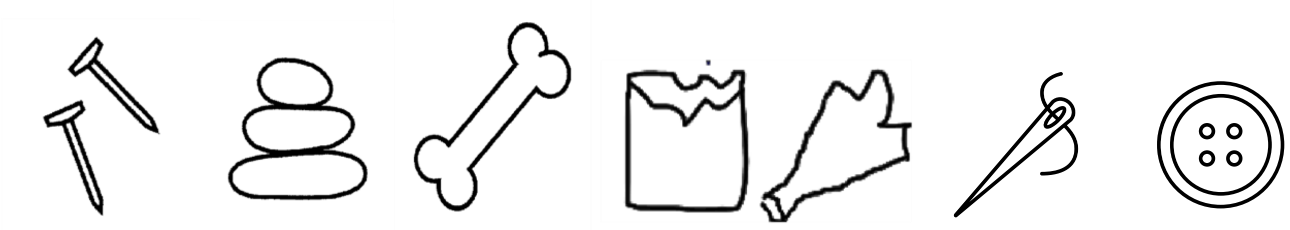

Adegoye, G.A, 2022

CHEMICAL CONTAMINANTS OR HAZARDS

Chemical contaminants or hazards are any substance in liquid, solid, or gaseous form that can cause danger or harm to humans when present in food. Examples are pesticides, sanitizers, cleaning substances, disinfectants, chemical (mercury, lead, detergents or soap), smoke, and gas.

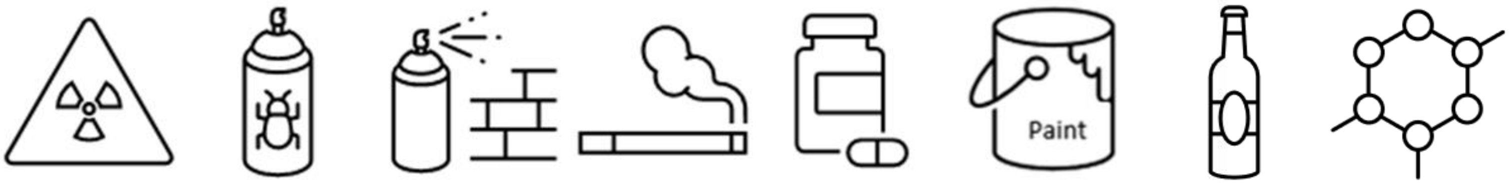

Adegoye, G.A, 2022

BIOLOGICAL CARRIERS OF DISEASES

Animals; pests, and insects/ flies e.g., houseflies, cockroaches are biological carriers of germs.

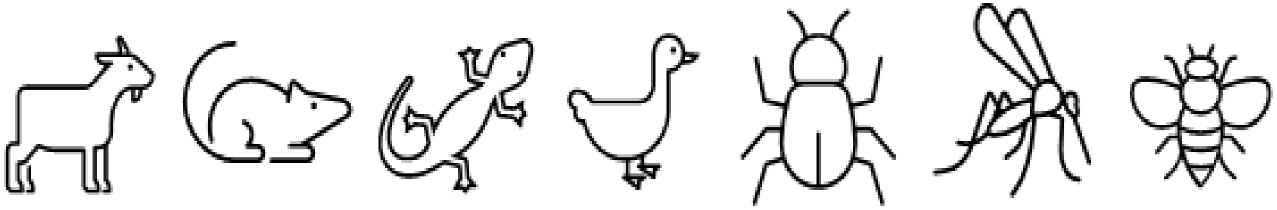

Adegoye, G.A, 2022

How do harmful agents get into fish products?

- Dirty hands containing germs

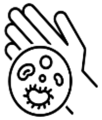

- Dirty plates, knives, buckets, or cutting slabs

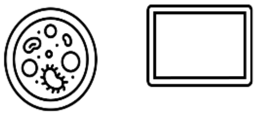

- Dirty and polluted water and ingredients

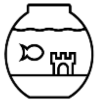

- Animals, insects, and birds

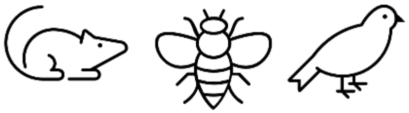

- Dirty floors and surroundings, uncovered waste bins

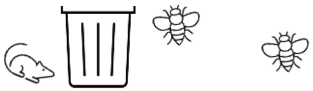

- Dirty clothes, napkins, handkerchief, and aprons

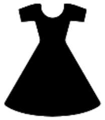

- Sick person (fish handler)

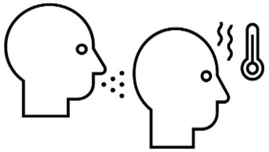

FEED THE FUTURE
The U.S. Government's Global Hunger & Food Security Initiative

FISH TOXINS AND POISONS

Fish toxins: Aflatoxin (mold growth on dried fish), Ciguatoxin (found in some marine algae.)

Chemicals poisons: like polyaromatic hydrocarbons, mercury, and lead.

Common symptoms of toxins OR poisons:

Dizziness	Numbness
Sweating	Vomiting
Headache	Confusion or memory loss
Tingling	Abdominal pain, coma, etc.

Effect of fish contamination and poisoning- See Module 3; Food safety.

USAID FROM THE AMERICAN PEOPLE

WorldFish

MISSISSIPPI STATE UNIVERSITY™ GLOBAL CENTER FOR AQUATIC FOOD SECURITY

FEED THE FUTURE
The U.S. Government's Global Hunger & Food Security Initiative

SAFETY GUIDELINE ON PESTICIDE USE AND WARNINGS

Fish is contaminated by pesticide from containers that are dropped into rivers, lakes, and seas.

Large fish are more likely to have a high level of pesticides in their flesh

Pesticides can build up to a toxic level in the body of the consumer

Small fish may be safer for children to reduce the risk of poisoning

Do not spray pesticide directly on raw or processed fish to kill germs, flies, or insects

Keep fish products safe from pesticide exposure.

Spray your shop before going home, after the day's sales if you must.

Take pesticides away from children's reach

USAID FROM THE AMERICAN PEOPLE
WorldFish
MISSISSIPPI STATE UNIVERSITY™ GLOBAL CENTER FOR AQUATIC FOOD SECURITY

FEED THE FUTURE
The U.S. Government's Global Hunger & Food Security Initiative

HOW TO STOP OR PREVENT HARMFUL SUBSTANCE FROM GETTING INTO FISH PRODUCTS

Wash your hands
Use clean water

Buy good raw material

Cook the fish properly

Personal hygiene

Allow proper cooling before packaging

Separate raw fish from processed fish

Reheat smoked fish in 2-3 days or once a week

USAID FROM THE AMERICAN PEOPLE

WorldFish

MISSISSIPPI STATE UNIVERSITY™
GLOBAL CENTER FOR AQUATIC FOOD SECURITY

FEED THE FUTURE
The U.S. Government's Global Hunger & Food Security Initiative

HOW TO HANDLE AN OPEN WOUND

Open wounds and cuts are sources of biological contaminants. They can be very dangerous

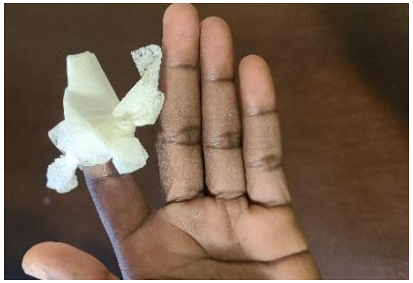

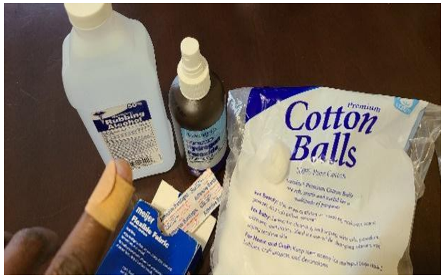

1. Wash your hands immediately with clean water
2. Do not leak the blood or stop the flow by putting it in your mouth
3. Apply pressure on the site of the wound to stop the blood flow
4. Apply disinfectant like methylated spirit, or hydrogen peroxide
5. Cover all wounds with a waterproof bandage or adhesive (plaster)
6. Go to the hospital if the bleeding does not stop

USAID FROM THE AMERICAN PEOPLE
WorldFish
MISSISSIPPI STATE UNIVERSITY™ GLOBAL CENTER FOR AQUATIC FOOD SECURITY

FEED THE FUTURE
The U.S. Government's Global Hunger & Food Security Initiative

ADDITIONAL TIPS

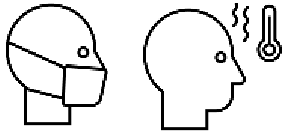

Check your body for fever, and stay at home if you are sick

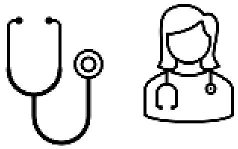

Call or visit your nurse or health caregiver for checkup

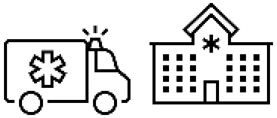

Visit hospital for proper care if you did not feel better

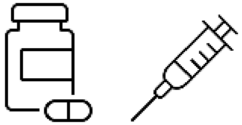

Use your pills as directed by the doctor and complete the treatment to prevent sickness from recuring.

If you have wounds use gloves while handling fish or spoons if possible

FAO/PAHO-WHO 2014, FAO

USAID FROM THE AMERICAN PEOPLE

WorldFish

MISSISSIPPI STATE UNIVERSITY™ GLOBAL CENTER FOR AQUATIC FOOD SECURITY

FEED THE FUTURE
The U.S. Government's Global Hunger & Food Security Initiative

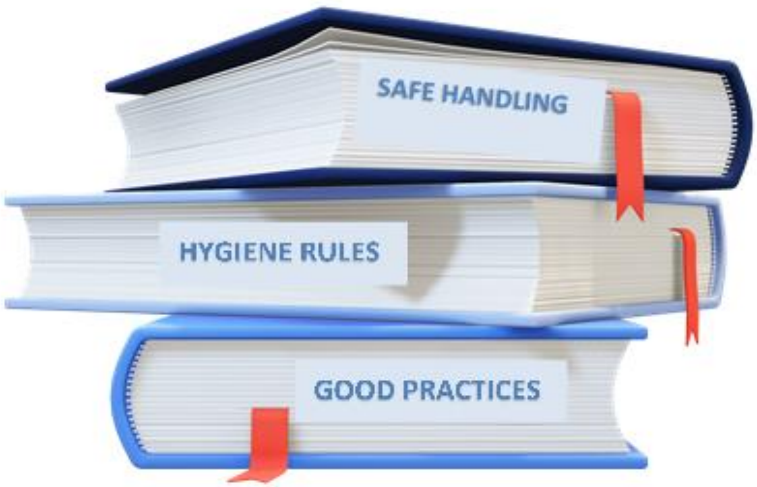

MODULE 6 HYGIENE RULES AND GOOD PRACTICES

Nourishing Nations Project

August 2021/Delta State, Nigeria

USAID FROM THE AMERICAN PEOPLE

WorldFish

MISSISSIPPI STATE UNIVERSITY GLOBAL CENTER FOR AQUATIC FOOD SECURITY

FEED THE FUTURE
The U.S. Government's Global Hunger & Food Security Initiative

SANITARY REQUIREMENTS OF FISH PROCESSING PREMISES

Clean fish processing environment (Sanitize and disinfect work area)

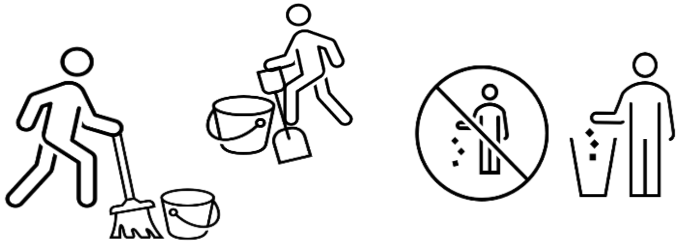

Clean water supply source for hand washing and fish processing.

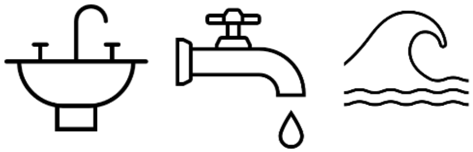

Clean toilet or sanitary improved latrine

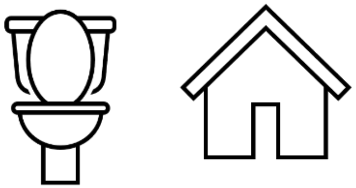

Free flowing channel for waste waster

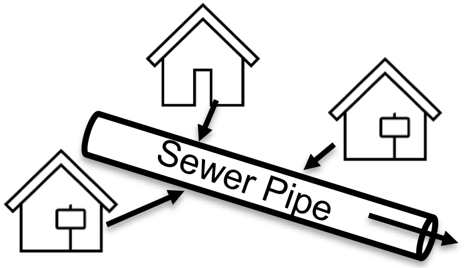

Federal Republic of Nigeria Official Gazette, 2016

USAID
FROM THE AMERICAN PEOPLE

WorldFish

MISSISSIPPI STATE UNIVERSITY™
GLOBAL CENTER FOR
AQUATIC FOOD SECURITY

FEED THE FUTURE
The U.S. Government's Global Hunger & Food Security Initiative

SANITARY REQUIREMENTS OF FISH PROCESSING PREMISES

Fish processing site or kitchen must **NOT** be close to a latrine or a dumping sites.

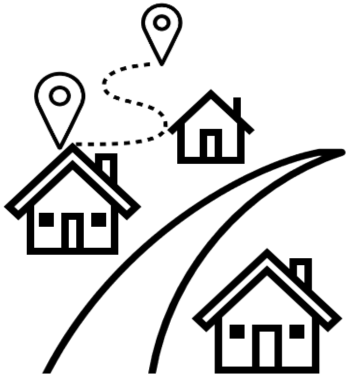

A sanitary waste bin with a well-fitted lid. Raise it 4 inches or 10 cm above the ground to keep rats, rodents, and flies out.

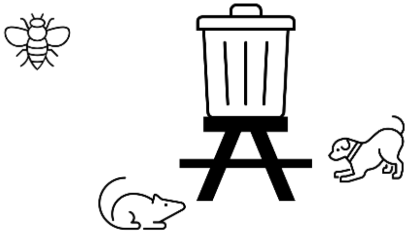

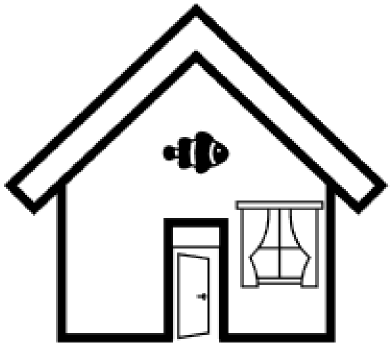

Fish processing kitchen must have enough openings for fresh air and light.

Federal Republic of Nigeria Official Gazette, 2016

USAID FROM THE AMERICAN PEOPLE
WorldFish
MISSISSIPPI STATE UNIVERSITY™ GLOBAL CENTER FOR AQUATIC FOOD SECURITY

OTHER SANITARY AND HEALTH REQUIREMENTS

A cold room or a cold storage facility

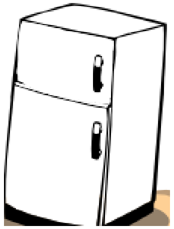

Adequate space and equipment for storage.

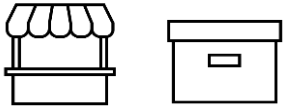

Fish processors must check their state of health two times a year, even if they do not appear sick.

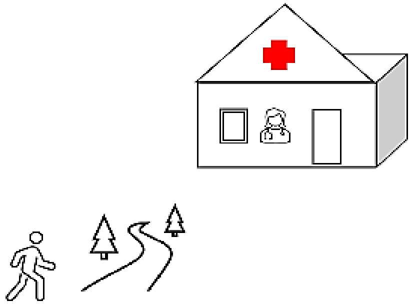

Fish processors should attend food safety training at least once a year.

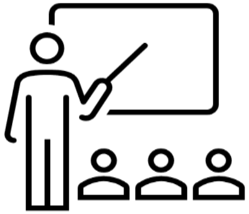

Federal Republic of Nigeria Official Gazette, 2016

FEED THE FUTURE
The U.S. Government's Global Hunger & Food Security Initiative

SANITARY RECOMMENDATIONS

Use material that will not allow rats to get into your kitchen or store. Repair broken floors, walls, and cracks to keep rodents out of the kitchen or shop. Call a trained pest manager.

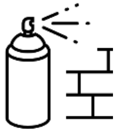

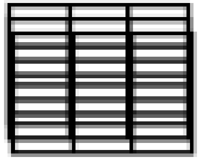

Use insect nets on the windows and doors to prevent flies.

Remove water-holding containers where mosquitoes can live.

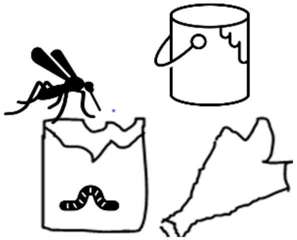

Remove cobwebs from the ceilings

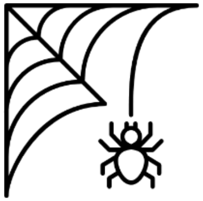

Federal Republic of Nigeria Official Gazette, 2016

USAID FROM THE AMERICAN PEOPLE

WorldFish

MISSISSIPPI STATE UNIVERSITY™ GLOBAL CENTER FOR AQUATIC FOOD SECURITY

SANITARY RECOMMENDATIONS

Sanitary disposal of spoiled fish, fish waste, and other domestic waste generated during processing.

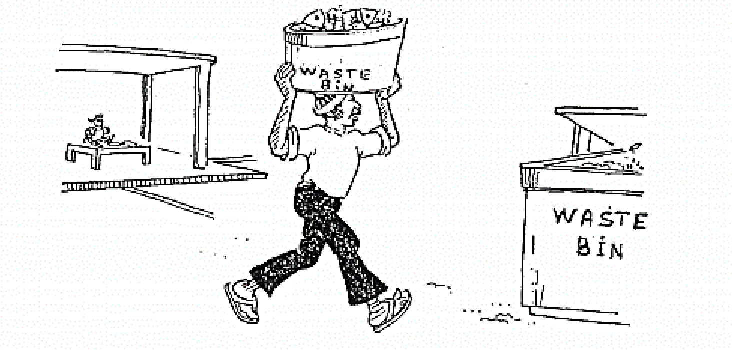

Wash the waste bin with soap and disinfectant.

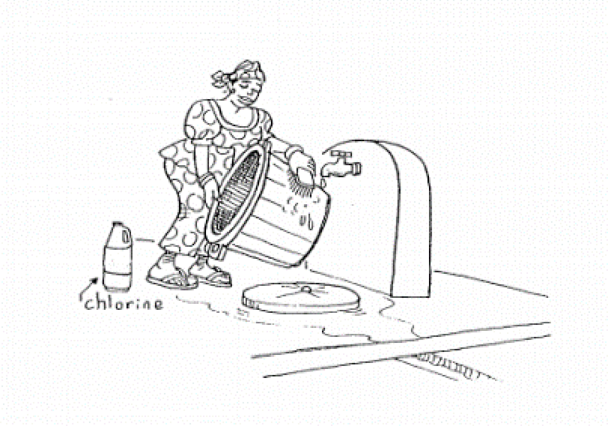

Diei, Y. and Ndiaye, O. 1998.

- Always wash your hands after cleaning the waste bin and before handling fish.

FEED THE FUTURE
The U.S. Government's Global Hunger & Food Security Initiative

ENVIRONMENTAL SANITATION

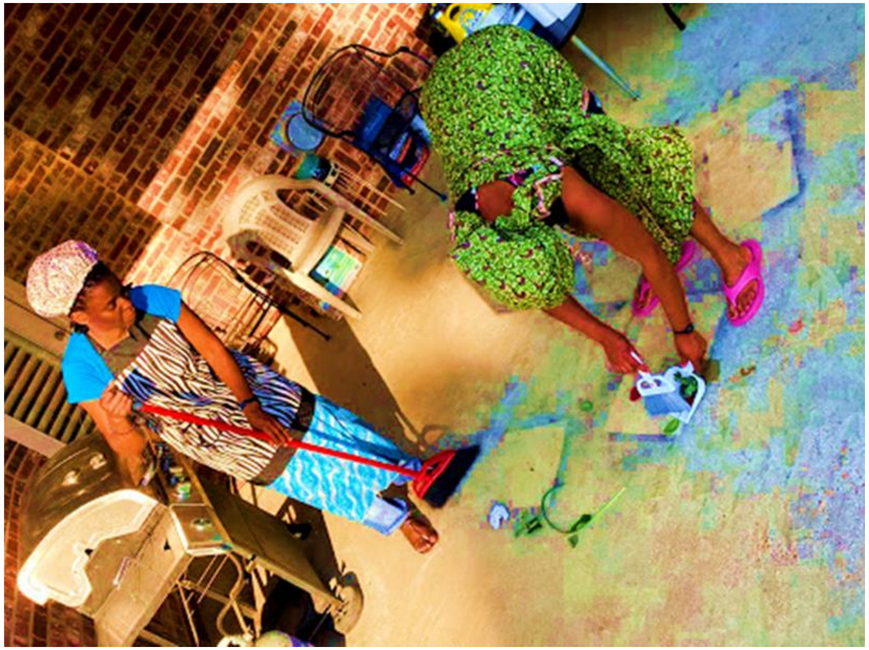
Photo by: Ayoola Babatunde. M

- Sweeping
- Mopping floors
- Wetting floors to prevent dust
- Washing floors and surfaces
- Picking trash
- Cleaning drainages
- Clearing dump sites
- Cutting overgrown weeds

USAID FROM THE AMERICAN PEOPLE

WorldFish

MISSISSIPPI STATE UNIVERSITY™ GLOBAL CENTER FOR AQUATIC FOOD SECURITY

FEED THE FUTURE
The U.S. Government's Global Hunger & Food Security Initiative

DISINFECTION (KILLING GERMS)

Disinfection is the act of killing germs or disease-causing agents in the environment, processing equipment, material, and surfaces where fish may likely be placed.

Instruction: Always use clean water, add 1 teaspoon of bleach or chlorine to 10 liters of water.

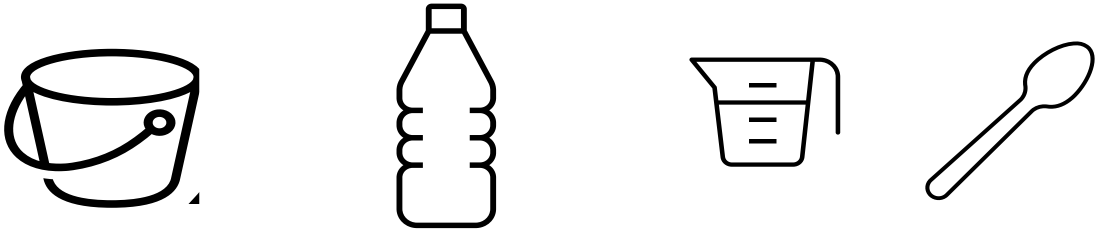

1bucket= 10 liters

Chlorine 1 liter

1 glass of water =150 ml

1 teaspoon = 10 ml

Adapted from Diei, Y. and Ndiaye, O. 1998.

USAID FROM THE AMERICAN PEOPLE

WorldFish

MISSISSIPPI STATE UNIVERSITY GLOBAL CENTER FOR AQUATIC FOOD SECURITY

GOOD PRACTICES

Good practices and a clean environment are required to have good result in the fish processing business.

Good Practices includes:

1. Good Hygiene Practices
2. Good Aquaculture Practice (in fisheries or aquatic food processing)
3. Good Transport Practices
4. Good Handling and Packaging Practices
5. Good Storage Practices

GOOD HYGIENE PRACTICES
GOOD AQUACULTURE PRACTICE

1. Good Hygiene practices (GHP)

- ❖ Hygiene and safety rules for fish handlers and processors (Module 3)
- ❖ Sanitary requirements of the fish processing premises

2. Good Aquaculture Practices

- ❖ Good practices in fish or seafood processing (Module 3 and 4)

FAO/PAHO-WHO 2014, FAO

GOOD TRANSPORT PRACTICES

i. During transportation, keep fish in ice flakes or crushed block ice.

ii. If transporting fish by road, it must be carried by protected and refrigerated trucks in good condition.

iii. Always keep the truck neat and clean. Also disinfect the truck to kill germs if needed

iv. Do not transport fish products in the same vehicle with:

- ✓ **Chemicals**
- ✓ **Pesticides**
- ✓ **Live animals**
- ✓ **Humans**

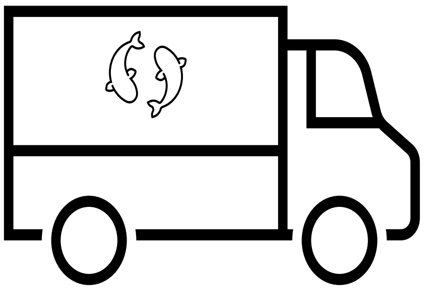

GOOD HANDLING AND PACKAGING PRACTICES

Good handling and packaging practices will protect all fish products from harmful substances and waste

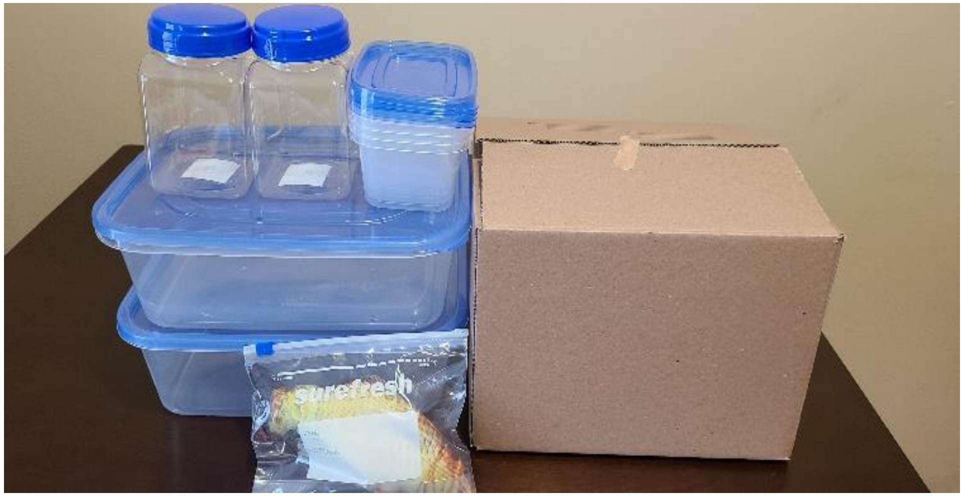

1. Fish products could be packed in carton, paper, or jute bags with plastic.
2. It can also be packed in a recyclable or reusable plastic (BPA free) container with a tight lid cover.

FEED THE FUTURE
The U.S. Government's Global Hunger & Food Security Initiative

GOOD HANDLING AND PACKAGING PRACTICES

Safe packaging tips:

1. Allow the processed fish to cool down before packaging.
2. Sort fish products before packaging
3. Handle them with care to avoid breaking them mistakenly.
4. Always place your packaging materials on the table or on a sanitized platform.
5. Use a solid, dry, clean, water -proof, and easy to handle material and stack.
6. Do not put too many fish or overload the packaging material.

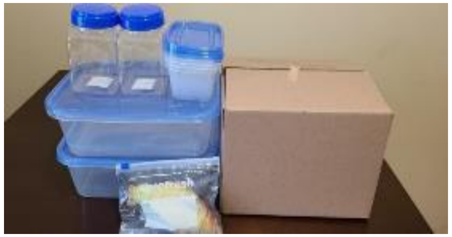

USAID FROM THE AMERICAN PEOPLE
WorldFish
MISSISSIPPI STATE UNIVERSITY™ GLOBAL CENTER FOR AQUATIC FOOD SECURITY

GOOD STORAGE PRACTICES

Storage of the final products is essential to prevent spoilage and waste. Good storage makes fish to last longer and stay safe in a good shape and form.

1. Store final products on shelves, wood planks, trays, trolleys, or in baskets.
2. Store in a dry, cool, well-aerated clean, and with natural lighting room.
3. Ensure a distance of **at least 15 cm (5.9 in) from the walls, ceiling, and ground level**.
4. Ensure the product is dry enough to prevent the growth of mold and keep fish product away from insects, rodents, or stray animals.

FAO/PAHO-WHO 2014, FAO

FEED THE FUTURE
The U.S. Government's Global Hunger & Food Security Initiative

GOOD STORAGE PRACTICES

1. Heat smoked fish regularly. During rainy season every 2-3 days, and once a week during the dry season

2. Always store in a clean condition, free of dirt, garbage or food waste.

3. Observe or check the products regularly and carefully.

4. Store finished fish products processed at different times or days separately and label.

USAID FROM THE AMERICAN PEOPLE

WorldFish

MISSISSIPPI STATE UNIVERSITY™ GLOBAL CENTER FOR AQUATIC FOOD SECURITY

FEED THE FUTURE
The U.S. Government's Global Hunger & Food Security Initiative

SAFETY INSTRUCTIONS ON CHEMICAL HANDLING

- Do not store raw or packaged processed fish products in chemical or pesticide containers/bags.
- Do not store chemicals such as detergents, or disinfectants in food storage containers to prevent accidental poisoning
- Pesticides are not preservatives, do not apply them to fish products.
- Chemicals must be stored separately from food storage areas
- Keep all chemicals, pesticide etc. away from the reach of children
- Wash your hands thoroughly after handling chemicals, or pesticides.

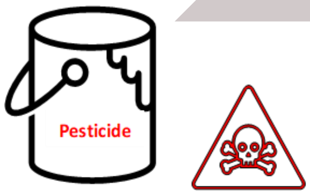

Adegoye et al., 2022

USAID FROM THE AMERICAN PEOPLE
WorldFish
MISSISSIPPI STATE UNIVERSITY GLOBAL CENTER FOR AQUATIC FOOD SECURITY

FEED THE FUTURE
The U.S. Government's Global Hunger & Food Security Initiative

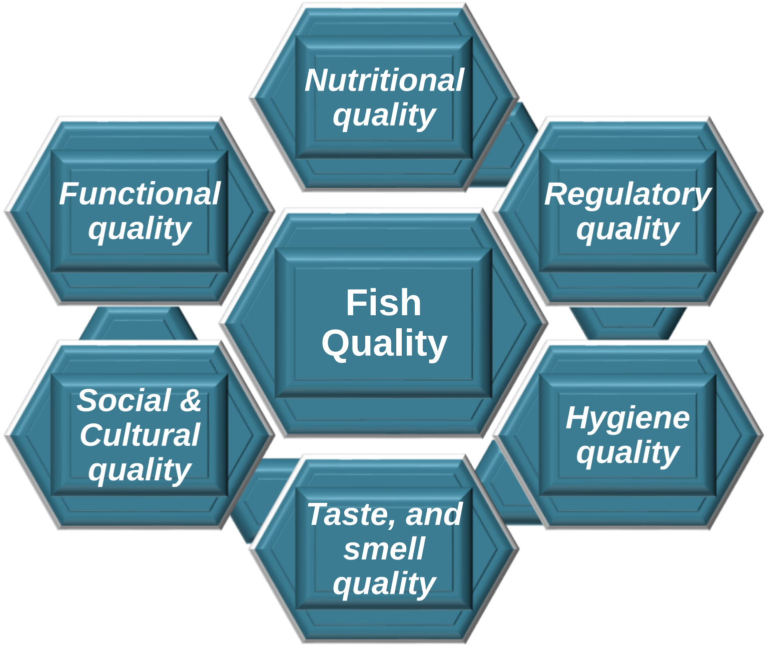

MODULE 7 ECONOMIC AND NUTRITION BENEFITS OF QUALITY FISH PRODUCTS

Nourishing Nations Project

August 2021/Delta State, Nigeria

USAID FROM THE AMERICAN PEOPLE

WorldFish

MISSISSIPPI STATE UNIVERSITY™ GLOBAL CENTER FOR AQUATIC FOOD SECURITY

FEED THE FUTURE
The U.S. Government's Global Hunger & Food Security Initiative

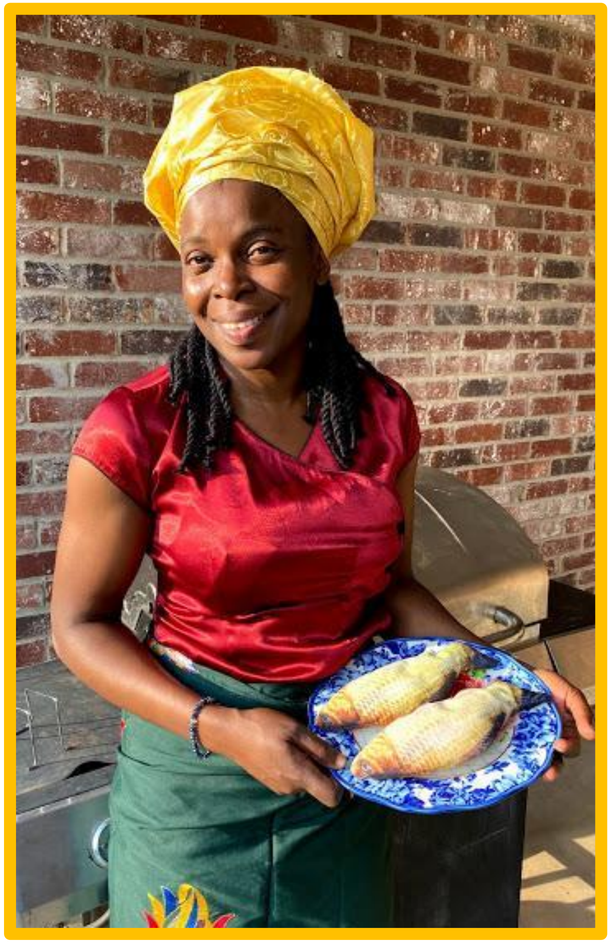

Photo by: Ayoola, Babatunde M

The better the quality,
The better your income,
The better your life.

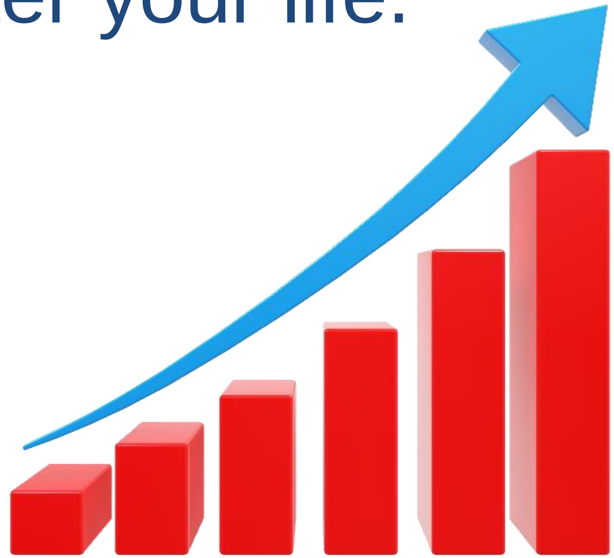

USAID FROM THE AMERICAN PEOPLE

WorldFish

MISSISSIPPI STATE UNIVERSITY™ GLOBAL CENTER FOR AQUATIC FOOD SECURITY

FISH QUALITY

Quality fish can be described as a fish product that is free from any harmful materials, or germs, and meets customer's satisfaction.

How to know a quality fish product

1. Has or retains the nutrient after processing
2. Meets the processing procedures and standards
3. Prepared in a clean and hygienic condition
4. Tastes and smells good
5. Appeals to the customers and is attractive and acceptable
6. Stored and transported under the right and safe condition

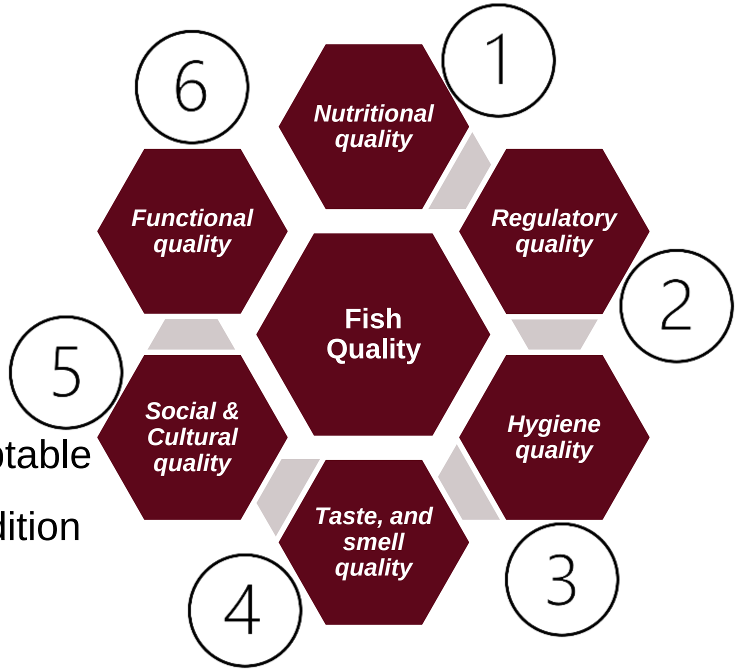

Adegoye 2022, Adapted from Cailliau, B. 2013

FEED THE FUTURE
The U.S. Government's Global Hunger & Food Security Initiative

HOW CAN QUALITY FISH PRODUCT IMPACT YOUR INCOME AND WELLBEING

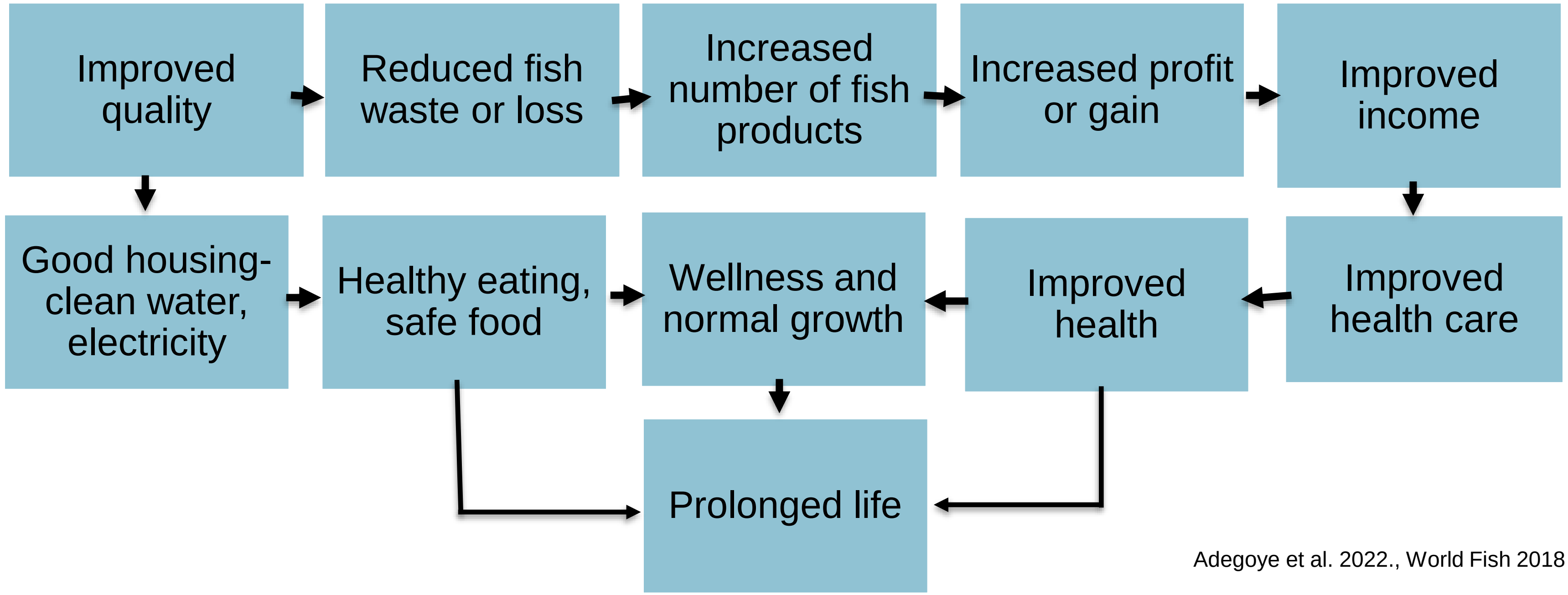

Adegoye et al. 2022., World Fish 2018

USAID FROM THE AMERICAN PEOPLE

WorldFish

MISSISSIPPI STATE UNIVERSITY™ GLOBAL CENTER FOR AQUATIC FOOD SECURITY

HOW TO REDUCE FISH WASTE

Reduce the price of the older fish products in stock.

First-in-first-out: Arrange the fish products with the closest best use by date in the front line of the shelf or desk.

Buy only the quantity you can process, preserve, and store.

Always use good packaging materials such as cartons, jute bags, and reusable plastics.

Ensure sanitary disposal of fish waste generated on daily basis.

NUTRITION AND HEALTH BENEFITS OF QUALITY AND SAFE FISH PRODUCT

Quality fish product:

1. Provide enough quantity and quality of nutrients needed for brain development and normal growth in children and pregnant women.
2. Lowers the risk of foodborne illnesses
3. Lowers the rate of visiting hospitals due to unsafe fish consumption.
4. Lowers the risk of food poisoning and foodborne illnesses
5. Reduces hospital bills due to foodborne illnesses
6. Lowers the risk of exposure to harmful substances
7. Improves nutrition and wellbeing.

Troell et al, 2019

FEED THE FUTURE
The U.S. Government's Global Hunger & Food Security Initiative

ECONOMIC BENEFITS OF QUALITY FISH PRODUCTS

Quality fish products will attract more buyers.	Reduction in post-harvest and post-farmgate loss	Reduce fish waste and spoilage	Fish will be available to consumers year round
New business opportunity	Reduce the economic loss and hospital bills due to foodborne illness, food poisoning.	New job opportunity for youths and women – economic empowerment	Canned fish can offer both nutrition and economic support.

World Fish 2018

USAID FROM THE AMERICAN PEOPLE

WorldFish

MISSISSIPPI STATE UNIVERSITY™ GLOBAL CENTER FOR AQUATIC FOOD SECURITY

HOW TO RECOGNIZE SPOILED OR POOR-QUALITY FISH TO AVOID ECONOMIC LOSS AND WASTE

- Dropped scale
- Presence of worms or insect larva, mold, and black spots
- Presence of offensive odor
- Sunk eyes
- Dark brown gills
- Flabby skin
- Attract flies

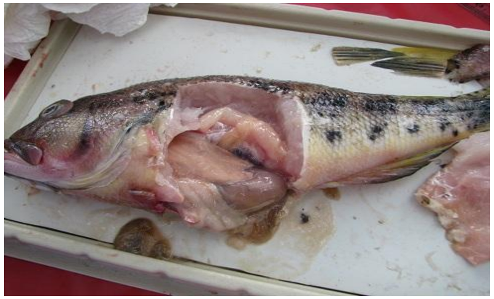

Photo source: internet

- Rodent bites and rat droppings
- Thawed fish
- Wound, injury, or bruise on fish
- Bad taste (cooked or processed product)

For canned fish;

- check for leakages,
- expiration,
- dent, bulging, and rusting on the can

AUTHORS

1. Adegoye, Grace Adeola, D.D, MEH, REHO, LEHO.
Ph.D. Candidate,
Department of Food Science, Nutrition, and Health Promotion.
Mississippi State University.

2. Tolar-Peterson, Terezie, EdD, RD, LD, FAND
Associate Professor,
Department of Food Science, Nutrition, and Health Promotion.
Mississippi State University.

ACKNOWLEDGMENTS

We appreciate the contribution of all the experts from various field in validating this educational material. Thanks to Professor Moses. A. Omishakin; Bamidele.B. Oni, (M.Sc. REHO, LEHO); Nicole, Reeder (M.Sc., R.D); Jasmine, Hendrix (M.Sc.); Dr. Rahel Matthew; Professor Juan. L. Silva; Professor Henrietta, N. Ene-Obong; and Dr. Joseph, N. Nuntah.

Drawings, images, infographics, and pictures are credited to Adegoye, Grace. A. unless otherwise stated.

Bibliographies are cited in the reference section [53-69] of the manuscript: Adegoye, G.A.; Tolar-Peterson, T.; Ene-Obong,N. H.; Nuntah, N.J.; Pasqualino, M.M.; Mathews, R.; Silva, J.L.; Cheng, W.-H.; Evans, M.W., Jr.; Pincus, L. Development and Validation of Nutrition and Food Safety Educational Material for Fish Processors in Nigeria. *Int. J. Environ. Res. Public Health* **2023**, *20*, x.

FEED THE FUTURE
The U.S. Government's Global Hunger & Food Security Initiative

BIBLIOGRAPHY

1. Modern Farmer. A Guide to the Roots and Tubers You Didn't Know You Loved (2016). https://modernfarmer.com/2016/01/roots-tubers-guide/ (accessed on March 12, 2021)

2. USDA MyPlate, Food and Nutrition Service, US Department of Agriculture. https://www.myplate.gov/ (accessed on January 13, 2021)

3. World Vegetable Center. Home Garden Toolkit https://avrdc.org/ (accessed on January 13, 2021)

4. Brown, J. E. (2016). *Nutrition through the life cycle*. Cengage Learning. 6th edition.

5. Centers for Disease Control and Prevention (CDC). Breastfeeding. https://www.cdc.gov/breastfeeding/about-breastfeeding/ (accessed on January 19, 2021)

6. Centers for Disease Control and Prevention (CDC). Public Health Image Library (PHIL) ID: 6901 https://phil.cdc.gov/details.aspx?pid=6902 (accessed on January 19, 2021)

7. Diei Y., and O. Ndiaye., Guide to proper artisanal fish handling and processing practices. 1998. Programme for the Integrated Development of Artisanal Fisheries in West Africa Programme (IDAF), Cotonou, Benin, 35p. IDAF/WP/129. http://www.fao.org/3/an685e/an685e.pdf . (accessed on January 14, 2021)

8. FAO/PAHO-WHO course for food handlers. Food and Agriculture Organization of the United Nations Regional Office for Asia and the Pacific Bangkok, 2014. Food Handler's Manual. https://iris.paho.org/handle/10665.2/34129. (accessed on January 13, 2021)

9. FAO. The principles and practice of fish handling and processing. http://www.fao.org/3/x5927e/x5927e01.htm. (accessed on February 3, 2021).

USAID FROM THE AMERICAN PEOPLE

MISSISSIPPI STATE UNIVERSITY™ GLOBAL CENTER FOR AQUATIC FOOD SECURITY

FEED THE FUTURE
The U.S. Government's Global Hunger & Food Security Initiative

BIBLIOGRAPHY

10. Servsafe Manager, 2017. 7th Edition, English. National Restaurant Association Educational Foundation.

11. Nigerian Smoked Fish Market Potential. https://www.meat-machinery.com/meat-processing-insight/nigerian-smoked-fish-market-potential.html (accessed on January 14, 2021).

12. Federal Republic of Nigeria Official Gazette: No. 27 Lagos, 15th February 2016. Vol.103. Short Title: National Environmental Health Practice Regulations, 2016, Page B223-266. (accessed on February 26, 2021).

13. Cailliau, Béatrice. "Hygiène et sécurité alimentaire." *L'Aide-Soignante* 27, no. 148 (2013): 25-29.https://doi.org/10.1016/j.aidsoi.2013.04.009 (accessed on March 24, 2021)

14. Troell, Max, Malin Jonell, and Beatrice Crona. "The role of seafood in sustainable and healthy diets." *The EAT-Lancet Commission report Through a Blue Lens. Stockholm: The Beijer Institute.* 2019. Pg 1-24. (accessed on March 24, 2021)

15. WorldFish Nigeria Strategy 2018–2022. *WorldFish. Strategy: 2018-09.*, 1–16. http://pubs.iclarm.net/resource_centre/2018-09.pdf (accessed on January 5, 2021)

16. Adegoye, Grace. A, Tolar-Peterson, Terezie, & Pincus, Lauren. (2022). Nutrition Education, Food Safety, and Safe Fish Handling Practice Guide for Fish Processors- Facilitators Guide. Zenodo. https://doi.org/10.5281/zenodo.7411946

17. Adegoye, G.A. Improving Nutrition and Food Safety Knowledge of Small-Scale Fish Processors in Delta State, Nigeria. Ph.D. Thesis, Mississippi State University, Starkville, MI, USA, 2022.

USAID
FROM THE AMERICAN PEOPLE

MISSISSIPPI STATE UNIVERSITY™
GLOBAL CENTER FOR
AQUATIC FOOD SECURITY
